# Novel PE‐DT-fusion toxin enhances internalization and target-dependently restores the cytotoxicity of recombinant immunotoxins

**DOI:** 10.3389/fphar.2026.1842007

**Published:** 2026-08-14

**Authors:** Claudia Fischer, Kerstin Wendland, Anna Ammon, Franziska Gsottberger, Lisa Mellenthin, Srdjan Petkovic, Andreas Mackensen, Fabian Müller

**Affiliations:** 1 Department of Internal Medicine 5-Hematology and Oncology, Friedrich Alexander University Erlangen-Nuremberg (FAU) and Universitätsklinikum Erlangen, Erlangen, Germany; 2 Bavarian Center for Cancer Research (BZKF), Erlangen, Germany; 3 Laboratory of Translational Immuno-Oncology, Department of Biomedicine, University and University Hospital Basel, Basel, Switzerland; 4 Deutsches Zentrum für Immuntherapie (DZI), Friedrich Alexander University Erlangen-Nuremberg and Universitätsklinikum Erlangen, Erlangen, Germany

**Keywords:** cytotoxicity, diphtheria toxin, internalization, *pseudomonas* exotoxin, recombinant immunotoxins, solid and hematological cancers, vesicular trafficking

## Abstract

**Background:**

Recombinant immunotoxins (RITs) are fusion proteins of a targeting domain, such as an antibody fragment, and a truncated toxin, including *Pseudomonas* exotoxin A (PE) or diphtheria toxin (DT). Limiting their possible fusion partners, the targeting domain of DT is typically fused C-terminally and that of PE is fused N-terminally. Among other factors, the activity of immunotoxins depends on the target antigen and the target-specific intracellular trafficking. Because a novel anti-CD138-PE immunotoxin was inactive against multiple myeloma, we hypothesized that rational toxin design would improve trafficking and, thus, cytotoxicity.

**Methods:**

We, therefore, generated several variants with distinct domain sequences including the catalytic (C) units of PE and DT, the furin-cleavage site of PE (fu), the transport (T) domain of DT, and the ER retention sequence KDEL. The lead candidate—a fusion of PE and DT—was combined with antibodies against CD138, CD22, glypican-3, or mesothelin.

**Results:**

This final toxin moiety was internalized 4-fold more efficiently over time on average compared to PE-based RITs, regardless of the target antigen or cell type. Improved internalization of DT over PE did not depend on a specific domain of DT. Instead, it was competed dose-dependently by poly-D lysine (PDL), indicating a more unspecific charge effect of DT over PE. The improved internalization frequently translated to enhanced cytotoxicity. Finally, combinatorial treatment with actinomycin D demonstrated synergistic effects with the lead PE‐DT-fusion toxin.

**Conclusion:**

This novel and target-dependently more potent toxin moiety may provide a framework for future immunotoxin design, possibly widening the range of target antigens, including the ongoing efforts to enhance mesothelin- or CD138-targeting immunotoxins against cancer or autoimmune diseases.

## 1 Introduction

In recent years, there has been a substantial increase of highly advanced therapies for cancer (Schambach et al., 2023; Dagar et al., 2023; Alewine et al., 2015). While some of these achievements are still under clinical trials, several patients still experience relapse or refractory disease, thus highlighting a still-existing need of novel therapeutic concepts, including unique ways of inducing targeted cell death. Recombinant immunotoxins (RITs) are chimeric fusion proteins of a targeting domain, such as an antibody fragment or interleukin, and a ribotoxic moiety, such as diphtheria toxin (DT) or *Pseudomonas* exotoxin A (PE) derived from *Corynebacterium diphtheriae* and *Pseudomonas aeruginosa*, respectively. These toxins block protein synthesis, which induces cell death by a unique mode of action (Pastan et al., 2006). After binding the cell surface via the targeting domain, the immunotoxin is internalized. Once in the late endosome, the toxin domain is liberated from the binding domain by furin cleavage and then traverses the toxin-specific intracellular compartments before escaping to the cytosol (Murphy, 2011; Michalska and Wolf, 2015). In the cytosol, the toxin ADP-ribosylates eukaryotic elongation factor 2 (eEF2), leading to a blockage of the ribosome and, thus, a stalling of protein synthesis (Pastan et al., 2006). The Bcl-2 family member Mcl-1 has an anti-apoptotic function and is among the proteins that are rapidly degraded in arrested protein synthesis, which eventually leads to BAK-dependent intrinsic apoptosis through mitochondrial outer-membrane depolarization (Michalska and Wolf, 2015; Antignani et al., 2017).

Various RITs have been developed preclinically, of which three have received clinical approval (Alewine et al., 2015; Pastan et al., 2006; Hamamichi et al., 2020). The HA22-PE-based moxetumomab pasudotox that targets CD22 in B-cell malignancies is active against hairy cell leukemia, denileukin diftitox utilizes interleukin-2 (IL-2) fused to DT to target CD25 and is approved in the treatment of T-cell malignancies, and tagraxofusp-erzs, a fusion of IL-3 and DT, targets CD123 and is highly active against blastic plasmacytoid dendritic-cell neoplasms (BPDCNs) (Pastan et al., 2006; Hamamichi et al., 2020). Ongoing (pre-)clinical development includes RITs such as the nanobody–immunotoxin HN3 targeting glypican-3 (GPC3) that is especially found in hepatocellular carcinoma, SS1P-derivates targeting mesothelin, or CD22-targeting RITs with close similarity to HA22 (Alewine et al., 2015; Fleming et al., 2020). Despite several improvements, especially regarding its immunogenicity, SS1(P)-derivates against mesothelin expressed in a variety of cancers, including mesothelioma, breast, pancreatic, ovarian, or cervical cancers, unfortunately failed to achieve clinically meaningful efficacy (Pastan et al., 2006; Hagerty et al., 2020; Hollevoet et al., 2015). The target antigen CD138 (syndecan-1) is aberrantly expressed in multiple myeloma (MM) and has not been successfully used in RIT constructs. Among other reasons, a critical limitation of immunotoxins is insufficient efficacy against the target cells *in vitro*, which has prompted the exploration of alternative apoptosis-inducing toxins, including ricin, as a ribotoxin (Alewine et al., 2015; Ammon et al., 2022; Morgan et al., 2023; Narbona et al., 2023).

**FIGURE 1:**
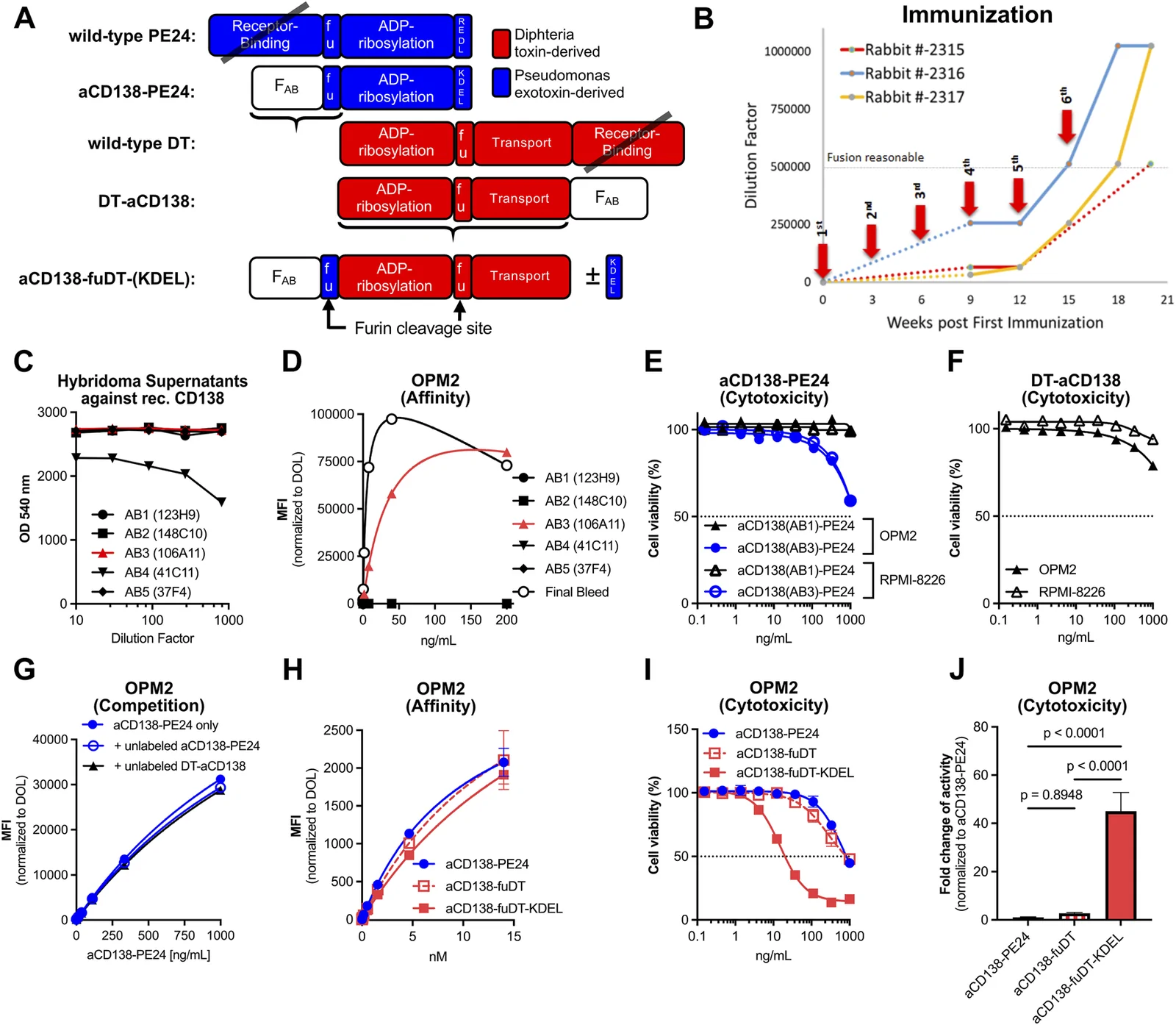
Development and comparative analysis of a novel RIT based on a fusion of PE and DT. **(A)** Schematic representation illustrating the stepwise evolution of the fusion protein aCD138-fuDT-KDEL, starting from wild-type PE24 (no transport domain), wild-type DT, and an aCD138 Fab. **(B)** Immunization of three rabbits as indicated with recombinant CD138, with dosing indicated by red arrows. **(C)** One of three representative ELISA of a serial dilution of the supernatant of five distinct hybridoma clones derived from rabbit #2316 against recombinant CD138. **(D)** MFI of OPM-2 cells stained with a 5-fold serial dilution of AlexaFluor647 (AF647)-labeled AB1-5, respectively, analyzed by flow cytometry and normalized to the degree of labeling (DOL) in mole dye per mole antibody. The DOL is the ratio of the dye concentration to the RIT concentration, representing how many dye molecules are bound to one antibody molecule. A representative of three independent experiments is shown. **(E)** Cell viability of OPM-2 and RPMI-8226 cells treated with aCD138(AB1)-PE24 and aCD138(AB3)-PE24 (referred to as aCD138-PE24) at varying concentrations. Cells were stained with 7-AAD, and the viability was measured by flow cytometry. Shown is a representative experiment of three similar results. **(F)** Cell viability of OPM-2 and RPMI-8226 cells following a 72-h incubation with a 3-fold serial dilution of DT-aCD138 at 37 °C. **(G)** MFI of OPM-2 cells treated with AF647-labeled aCD138-PE24 alone or competing with 80 ng/mL of either unlabeled aCD138-PE24 or DT-aCD138 for cell binding. Affinity was measured by flow cytometry and normalized to the DOL. **(H)** MFI of OPM-2 cells treated with AF647-labeled aCD138-PE24, aCD138-fuDT, or aCD138-fuDT-KDEL at 14 nM and normalized to the DOL in mole dye per mole RIT. **(I)** Cytotoxicity assay of OPM-2 cells treated with aCD138-PE24, aCD138-fuDT, or aCD138-fuDT-KDEL. Cell viability was determined using 7-AAD and measured by flow cytometry. One of three similar results is shown. **(J)** Fold-change of activity normalized to aCD138-PE24 calculated for OPM-2 cells using IC_50_ values pooled from three independent experiments with error bars as SD. Activity is defined as the inverse IC_50_. Statistical significances were determined by ordinary one-way analysis of variance (ANOVA) combined with Tukey’s multiple comparisons test. For **(D-I)**, non-linear regression analyses were performed to highlight the progression of cell binding or cell death depending on the RIT dose. Each data point represents either one **(C–F)** or the mean of two **(G–I)** technical replicates, with error bars as SD. The graphs are representative for either two **(E)** or three **(D,F–I)** independent experiments, respectively.

PE consists of the N-terminal receptor-binding domain, followed by the transport domain harboring the furin-cleavage site (fu) and the C-terminal ADP-ribosylation domain (Figure 1A). Flipped in the domain structure, DT consists of an N-terminal ADP-ribosylation domain, followed by the furin-cleavage site, the transport (T), and the C-terminal binding domain (Hwang et al., 1987; Choe et al., 1992). Up-to-date, generated immunotoxins generally maintained the naturally occurring order of domains, with DT-based immunotoxins typically using cytokines as their C-terminal-targeting moiety (Pastan et al., 2007; Allahyari et al., 2017), while PE-based toxins typically are N-terminally fused to antibody fragments (Pastan et al., 2007; Allahyari et al., 2017). Despite the reversed domain order, the toxin domain of DT and PE harbor similar domain structures and closely mimic their catalytic function (Kreitman, 2009). The intracellular transport of DT and PE, however, is vastly different (Murphy, 2011; Michalska and Wolf, 2015). After internalization, DT reaches the late endosome as its default target vesicle and establishes a pH-dependent membrane pore, through which it escapes to the cytosol (Donovan et al., 1981; Zalman and Wisnieski, 1984). PE, in contrast, traverses various intracellular compartments including the trans-Golgi network using the KDEL-receptor-dependent retrograde trafficking to the ER, where it escapes through the translocon (Michalska and Wolf, 2015).

Based on a surprisingly dysfunctional anti-CD138 immunotoxin, here, we developed a hypothesis-driven approach to a novel and uniquely structured toxin moiety. As a novelty, the lead DT-based toxin consisted of an N-terminal antibody fragment, followed by the furin-cleavage site of PE, the ADP-ribosylation domain, a second furin-cleavage site, and the DT transport domain and was further enhanced by a C-terminal KDEL sequence (Figure 1A). We compared toxin internalization and efficacy against various distinct target proteins and cancer cells *in vitro*, thus directly and indirectly elucidating novel mechanistic insight for future toxin designs.

## 2 Materials and methods

### 2.1 Cell lines

Cell lines OPM-2, RPMI-8226, KOPN8, JeKo-1, KB-3-1, OvCAR-3, Capan-1, Capan-2, Hep3B, and Huh-7 were maintained under standard conditions at 37 °C and 5% CO_2_. Suspension cells were cultivated in RPMI-1640 medium, IMDM, or McCoy’s 5A Medium (Gibco™, Thermo Fisher Scientific), while adherent cells were kept in DMEM (Gibco™, Thermo Fisher Scientific). Details regarding the specific supplements added into the media are provided in Supplementary Table S1.

### 2.2 Development of anti-CD138 antibodies

Antibody production was performed by GenScript. Rabbit splenocytes were fused with a secretory myeloma cell line and single-cloned, and the single clones were cultured in hybridoma medium supported by growth factors. The crude cell-culture supernatant was first screened against recombinant CD138 for binding using an ELISA. The top-five binders were sequenced, and single-clone supernatants were used for cellular binding assays. This supernatant was incubated on the OPM-2 myeloma cell line, and binding to the cell surface was analyzed by mouse anti rabbit-IgG-PE (Jackson ImmunoResearch) and visualized using the FACSCanto II flow cytometer. Data were analyzed using the FlowJo software v10.8.1.

### 2.3 Generation and purification of immunotoxins

To generate immunotoxins, plasmids from pre-existing RITs were used to create both new combinations with each other or with gene blocks ordered from IDT (IDT, Coralville, IA, United States) via restriction digesting and cloning (Supplementary Figure S1). The production and purification of the immunotoxins followed established protocols (Pastan et al., 2004). In summary, BL21(DE3) *E. coli* (NEB, Ipswich, MA, United States) were used for the individual expression of light (LC) and heavy chains (HC-toxin) of the disulfide-stabilized antigen-binding fragment (ds-Fab) of the recombinant immunotoxins under the control of a T7 promoter. After successful transformation, bacteria were amplified in 2 L of pre-heated TB medium under constant shaking (250 rpm at 37 °C) until an optical density of 2.0–3.0 at 600 nm was reached. Then, protein production was induced by adding 0.1 M Isopropyl β-D-1-thiogalactopyranoside (IPTG) via the T7 RNA polymerase, followed by 2 h of incubation at 37 °C and 250 rpm. The bacteria were harvested, and immunotoxin chains were recovered from the inclusion bodies using a disperser and lysozyme (10 mg/mL, Carl Roth) to disrupt the bacterial cell wall. To reduce endotoxin contamination, the inclusion bodies were washed thrice with 25% Triton X-100 and TES buffer (50 mM Tris-HCl, pH 8.0, 20 mM EDTA, and 100 mM NaCl) and thrice with TES buffer alone (Onda and Chames, 2012). Next, the RITs were solubilized and reduced at room temperature in GTE buffer (6 M guanidine HCl, 100 mM Tris-HCl, pH 8.0, and 20 mM EDTA) overnight, and the concentrations were adjusted. LC and HC were then combined and further reduced at room temperature with 1,4-dithioerythritol (DTE, Sigma-Aldrich) overnight. Refolding was carried out over 32 h at 4 °C in refolding buffer (100 mM TRIS, 0.5 M L-arginine, 1 mM EDTA, and 0.551 g/L L-glutathione; pH 9.5), followed by a 72-h dialysis at 4 °C in dialysis buffer (30 mM Tris-HCl, pH 7.5, and 100 mM urea). Finally, RITs were purified using an ÄKTApure chromatography system (Cytiva), first passing through two anion exchange columns (Q Sepharose Fast Flow and Capto HiRES, Cytiva) and subsequently through a size exclusion column (Superdex 75 Increase GL, Cytiva), as previously described, to further remove the misfolded proteins and impurities (Pastan et al., 2004). The final protein purity was monitored after every major production run using the ÄKTA system, and each purification step, along with the final products, was analyzed under both the reducing and non-reducing conditions by SDS-PAGE with Coomassie staining.

### 2.4 Cell assays

For *in vitro* binding, competition, and internalization measurements, RITs were labeled using the AlexaFluor647 (AF647) Antibody Labeling Kit (Thermo Fisher Scientific, Waltham, MA, United States) following the manufacturer’s instructions. The degree of labeling (DOL) was determined using the following equation:

$$\text{DOL in moles dye per mole RIT}=\frac{c\left(\text{AF}647\;\text{Dye}\;\text{in}\;µM\right)}{c\left(\text{RIT}\;\text{in}\;µM\right)}.$$

For binding assays, 2.5 x 10^5^ cells per well were treated with a 3-fold serial dilution of immunotoxins, starting with 1000 ng/mL or 14 nM in case of varying molecular masses. Cells were incubated for 1 h at 4 °C in FACS buffer (PBS supplemented with 0.1% sodium azide and 5% FBS), and affinity was determined via flow cytometry. To test the competition, 80 ng/mL of unlabeled competing immunotoxin was added to the binding assay.

For internalization assays with suspension cells, 2.5 x 10^5^ cells per well were incubated with 14 nM of RITs (HN3-RITs: 25 nM, fragmented aCD138-RITs: 15 nM) for various time points (0.5 h, 1 h, 2 h, and 3 h) at 37 °C. Untreated cells served as the controls. After reaching the respective time-point, samples were washed and stripped of surface-bound RITs using a stripping buffer (150 mM glycine and 50 mM NaCl, pH 2.7). For adherent cell lines, cells were plated 24 h prior and RITs were added at the respective time-points. After 3 h, all cells were washed at once, stripped, and detached using 5% trypsin at 37 °C. The amount of internalized RIT was analyzed via flow cytometry.

To assess the influence of lysines on RIT internalization, the immunotoxins were pre-incubated with a 2-fold serial dilution of the positively charged amino acid polymer poly-D lysine (PDL) for 30 min at 37 °C. Afterward, an internalization assay with a single incubation period of 90 min was performed as described above. A serial dilution of PDL only was handled accordingly, which served as a control.

For cell apoptosis and proliferation arrest assays, 2x 10^4^ target cells per well (5 x 10^3^ for the proliferation assay) were treated with a 3-fold serial dilution of RITs starting with a maximum concentration of 1000 ng/mL (100 ng/mL for KOPN8). Cells were incubated for 72 h at 37 °C and 5% CO_2_. Afterward, cells were either stained with the viability dye 7-amino actinomycin D (7-AAD, BioLegend) and analyzed via flow cytometry (Philpott et al., 1996) or incubated for further 2 h with a mixture of the non-toxic tetrazolium salt WST-8 (final c: 0.5 mM) and the electron carrier 1-methoxy-5-methylphenazinium methyl sulfate (1-methoxy-PMS, final c: 20 µM). In the latter assay, WST-8 is reduced by cellular dehydrogenases in the presence of 1-methoxy-PMS to form an orange formazan dye, the amount of which is directly proportional to the number of viable cells and can be quantified (Müller et al., 2018; Dusinska et al., 2017). Absorbance was measured using a SpectraMax® M3 Microplate reader (VWR) at 460 nm. Untreated controls were used for normalization. For cell apoptosis measurements with adherent cells, cells were plated at least 4 h prior to the start of the 72-h incubation period.

To determine the effect of a dual treatment strategy of RIT combined with actinomycin D, apoptosis measurements were performed as described above. Samples included single treatment controls of RIT or actinomycin D alone, with the addition of 10 ng/mL actinomycin D to the dual treatment samples.

### 2.5 Statistical analysis

Statistical testing was performed with GraphPad Prism version 9.4.1. Ratio paired Student`s t-tests, ordinary ANOVA combined with Tukey`s multiple comparison tests, or two-way ANOVA combined with Šídák’s multiple comparisons tests were used as indicated in the figure legends. Non-linear regression analyses were performed to predict the course of binding, internalization, and activity of RITs. The mean fluorescence intensity (MFI) was calculated using FlowJo v10.8.1.

## 3 Results

### 3.1 Development and characterization of a novel PE-DT-fusion RIT targeting CD138

Starting with the generally accepted hypothesis that multiple myeloma has a very high demand on the protein production machinery, we hypothesized that this cancer may be exclusively sensitive to a perturbation of its protein synthesis. Thus, we aimed at generating a CD138-targeting PE-based RIT (Carey, 1997). We immunized three rabbits with a recombinant extracellular domain of the glycoprotein CD138 (Supplementary Figure S2). With increasing numbers of immunizations, the sera of all three rabbits showed a step-wise increase in reactivity against CD138 (Figure 1B). Rabbit #2316 was used for spleen B-cell isolation and the generation of hybridomas, of which the five strongest binders were sequenced and further characterized. All five clones showed strong binding to CD138 that was recombinantly produced in bacteria (Figure 1C). Possibly due to the glycosylation of CD138 in eukaryotic cells, only antibody 3 (AB3) and, on a much lower level, AB1 possessed affinity to CD138 expressed on the cell surface of the MM cell line OPM-2 (Figure 1D). We then constructed immunotoxins that consisted of the fragment antigen-binding region (Fab) of high-affinity AB3 or lower affinity AB1 and the truncated bacterial toxin PE24, respectively (Figure 1A; Supplementary Figures S3A,B) (Weldon et al., 2009; Müller et al., 2018). AB3-based aCD138-PE24 showed some dose-dependent cytotoxicity against OPM-2 and RPMI-8226 cells at the highest concentrations, while the lower affinity aCD138(AB1)-PE24 was inactive (Figure 1E).

To better understand the unexpectedly poor efficacy, we tested the rate of internalization of the AB3-based aCD138-PE24 and found steep and linear internalization of the immunotoxin in both the OPM-2 and RPMI-8226 cells (Supplementary Figure S3C). This indicated that there was a step after internalization that was dysfunctional when targeting CD138 on MM via PE. Thus, we exchanged the toxin moiety from PE to DT. In line with the physiologic DT, the targeting moiety was attached C-terminally (Figure 1A). The protein was expressed and showed good purity (Supplementary Figure S4). However, DT-aCD138 also proved to be inactive against OPM-2 and RPMI-8226 cells (Figure 1F). We hypothesized that the DT may interfere with C-terminal antibody refolding and tested the affinity of DT-aCD138 in competition with AlexaFluor647(AF647)-labeled aCD138-PE24. Even at the highest concentrations of DT-aCD138, aCD138-PE24 binding to OPM-2 was not competed, indicating no or poor binding of DT-aCD138 to the target antigen (Figure 1G). In line with these results, a DT-based immunotoxin using the anti-FMS-like tyrosine kinase 3 antibody EB10, DT-EB10, failed to compete full length (fl-)EB10-PE from binding to the target cells KOPN8 and RS4;11 (Supplementary Figures S5A-C), supporting that an antibody fragment fused C-terminally to DT may not lead to high-affinity immunotoxins. We next hypothesized that the antibody would have to be attached at the N-terminal end of DT to allow for proper folding and binding of the antibody fragment. To still achieve liberation of DT from the antibody moiety, we designed a fusion RIT termed aCD138-fuDT-KDEL with an N-terminal aCD138 Fab followed by the furin-cleavage site of PE24 and the C- and T-domains of DT. Additionally, the ER retention sequence KDEL was added C-terminally to influence intracellular trafficking (Figure 1A) (Kreitman and Pastan, 1995; Jackson et al., 1999). Supporting a sterical interaction of N-terminal DT and the antibody, all three immunotoxins now exhibited similar affinity toward MM cells, indicating that the C-terminal antibody fragment also performed well in the novel DT toxins once attached by the PE-linker (Figure 1H). Intriguingly, aCD138-fuDT-KDEL demonstrated a 40-fold higher cytotoxicity toward OPM-2 and RPMI-8226 cells than aCD138-PE24 or aCD138-fuDT (Figures 1I,J; Supplementary Table S2; Supplementary Figure S5D). Because the affinity of all three immunotoxins was similar, the higher cytotoxicity of aCD138-fuDT-KDEL over the non-KDEL variant or PE24 had to be mechanistically explained by an effect following target binding on the cell surface.

### 3.2 fuDT-KDEL-based immunotoxins show superior internalization

**FIGURE 2:**
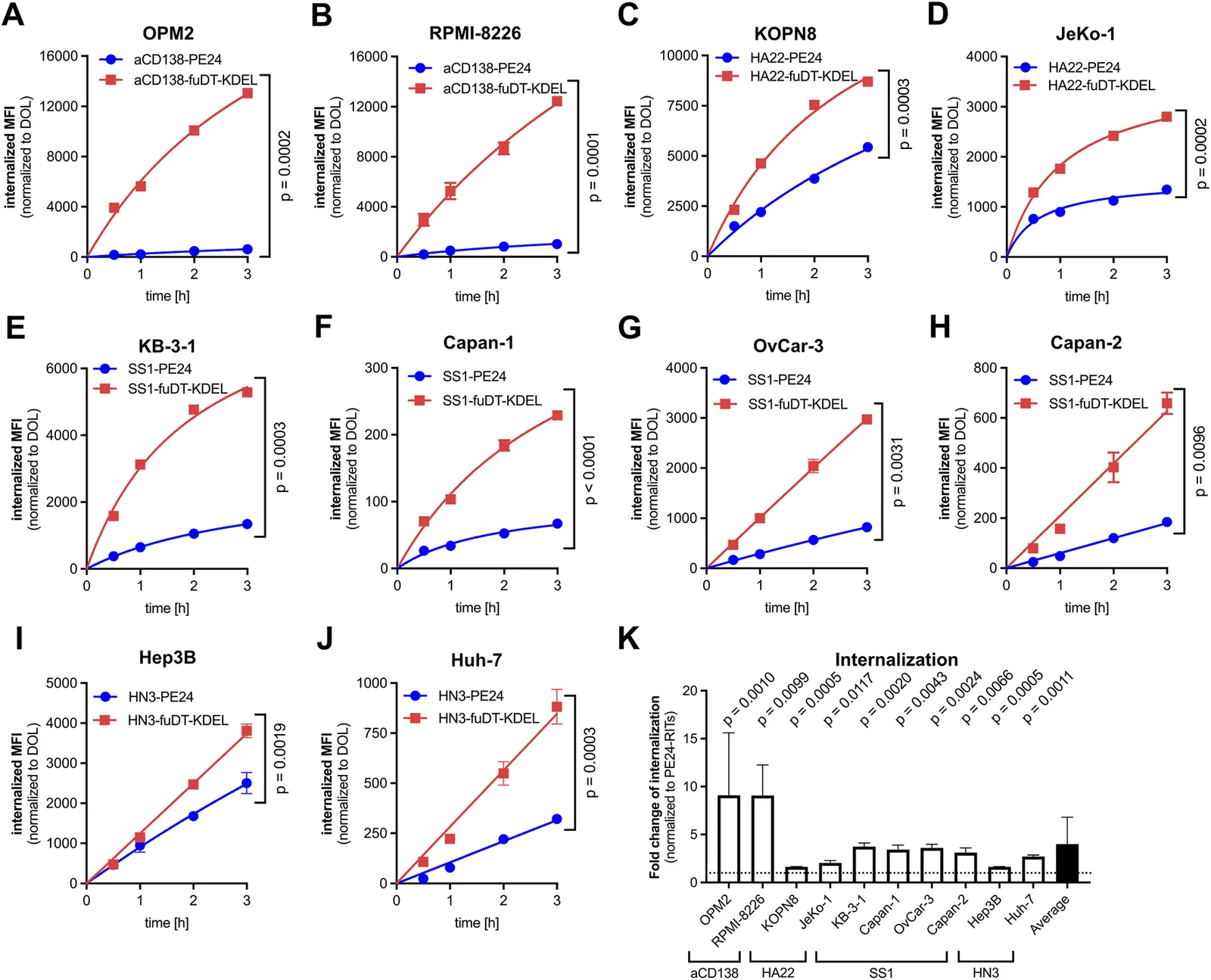
Target-independent enhancement of internalization of fuDT-KDEL immunotoxins. Continuous exposure of the indicated AlexaFluor647-labeld immunotoxins leads to distinct internalization, as determined after surface stripping of not yet internalized protein by flow cytometry of **(A)** OPM-2, **(B)** RPMI-8226, **(C)** KOPN8, **(D)** JeKo-1, **(E)** KB-3–1, **(F)** Capan-1, **(G)** OvCar-3, **(H)** Capan-2, **(I)** Hep3B, and **(J)** Huh-7 cell lines incubated with 14 nM of the respective immunotoxin and normalized to the DOL in mole dye per mole RIT. Non-linear regression analyses over time are shown; each dot represents the mean of two technical replicates, with error bars as SD. Statistical significances were calculated by two-way ANOVA combined with Šídák’s multiple comparisons test. Each graph is representative for three independent experiments. **(K)** Fold-change of internalization of fuDT-KDEL-based RITs at 3 h normalized to PE24-based RITs summarizing cumulative data from three independent experiments. Error is shown as SD, and statistical significances were calculated by ratio paired t-test.

To determine whether their distinct toxicity profile was due to internalization, we used the respective fluorochrome-conjugated variants of the generated RITs on OPM-2 or RPMI-8226 in a time-dependent internalization assay (Figures 2A,B). Interestingly, aCD138-fuDT-KDEL internalized significantly and continuously faster than PE24 for both cell lines. There was no difference in internalization between aCD138-fuDT and aCD138-fuDT-KDEL (Supplementary Figure S6A). To assess whether the advantage of fuDT-KDEL was a general effect, we tested distinct target proteins, including CD22 (HA22) on B-cell malignancies (KOPN8 and JeKo-1); mesothelin (SS1) on cervical (KB-3-1), pancreatic (Capan-1, -2), or ovarian (OvCar-3) carcinoma cell lines; and glypican-3 (HN3) on liver cancer cell lines (Hep3B and Huh-7) (Fleming et al., 2020; Hollevoet et al., 2014; Wayne et al., 2014). RITs were expressed and found to have adequate purity to test their internalization and cytotoxicity (Supplementary Figure S6B-D2). Similar to aCD138, most PE24 and fuDT-KDEL toxins showed no significant differences in affinity toward their respective target cells (Figure 1H; Supplementary Figure S7A–I). Notable exceptions included HA22-fuDT-KDEL, which exhibited overall weaker binding to lymphoma cells than its PE24 counterpart (Supplementary Figures S7C,D), and SS1-fuDT-KDEL, which demonstrated significantly improved binding to KB-31 but not to OvCar-3 or Capan-2 cells (Supplementary Figures S7E-G). The maximum amount of RIT bound to the target cells corresponded to the reported antigen density of the respective cell lines, as reported in the literature (Supplementary Figure S7J) (Chu et al., 2024; Gao et al., 2015; Hagemann et al., 2019; Ha et al., 2013; Lee et al., 2012; Yu et al., 2019).

Despite minor differences in affinity, the fuDT-KDEL toxins consistently internalized substantially faster into all tested cells (Figures 2C–J; Supplementary Figure S8). The internalization followed two distinct kinetics. It showed a hyperbolic increase, indicating saturation over time in OPM-2, RPMI-8226, KOPN8, JeKo-1, KB-3-1, and Capan-1 cells (Figures 2A–F). In contrast, a linear increase without evident saturation within the first 3 hours of the internalization process was found in OvCar-3, Capan-2, Hep3B, and Huh-7 cells (Figures 2G–J). The average normalized fold-change of internalization at 3 hours was target- and cell-type-specific, with the highest average enhancements observed with aCD138 (OPM2/RPMI-8226: 9.1-fold), intermediate enhancement for SS1 (anti-mesothelin) (KB3-1: 3.7-fold, Capan-1: 3.4-fold, OvCar-3: 3.6-fold, Capan-2: 3.1-fold), and the lowest enhancement in HA22 (anti-CD22) (KOPN8: 1.6-fold, JeKo-1: 2.0-fold) or glypican-3 targeting (Hep3B: 1.6-fold and Huh-7: 2.7-fold). The average normalized fold-change of internalization at 3 hours over all the analyzed targets showed a substantial and highly significant 4-fold higher internalization of fuDT-KDEL compared with PE (Figure 2K; Supplementary Figure S8). Questioning whether improved internalization correlated with the amount of surface-bound molecule, we normalized the internalized to total RIT at 3 h and confirmed that more fuDT-KDEL per bound molecule was present inside the cell compared with PE24, supporting the improved internalization of the former (Supplementary Figure S9A). Although not analyzed for all targets, SS1-fuDT-KDEL was not internalized by mesothelin-negative OPM-2 cells, which is consistent with target antigen-specific internalization (Supplementary Figure S9B).

### 3.3 The transport domain of DT is important for superior internalization

**FIGURE 3:**
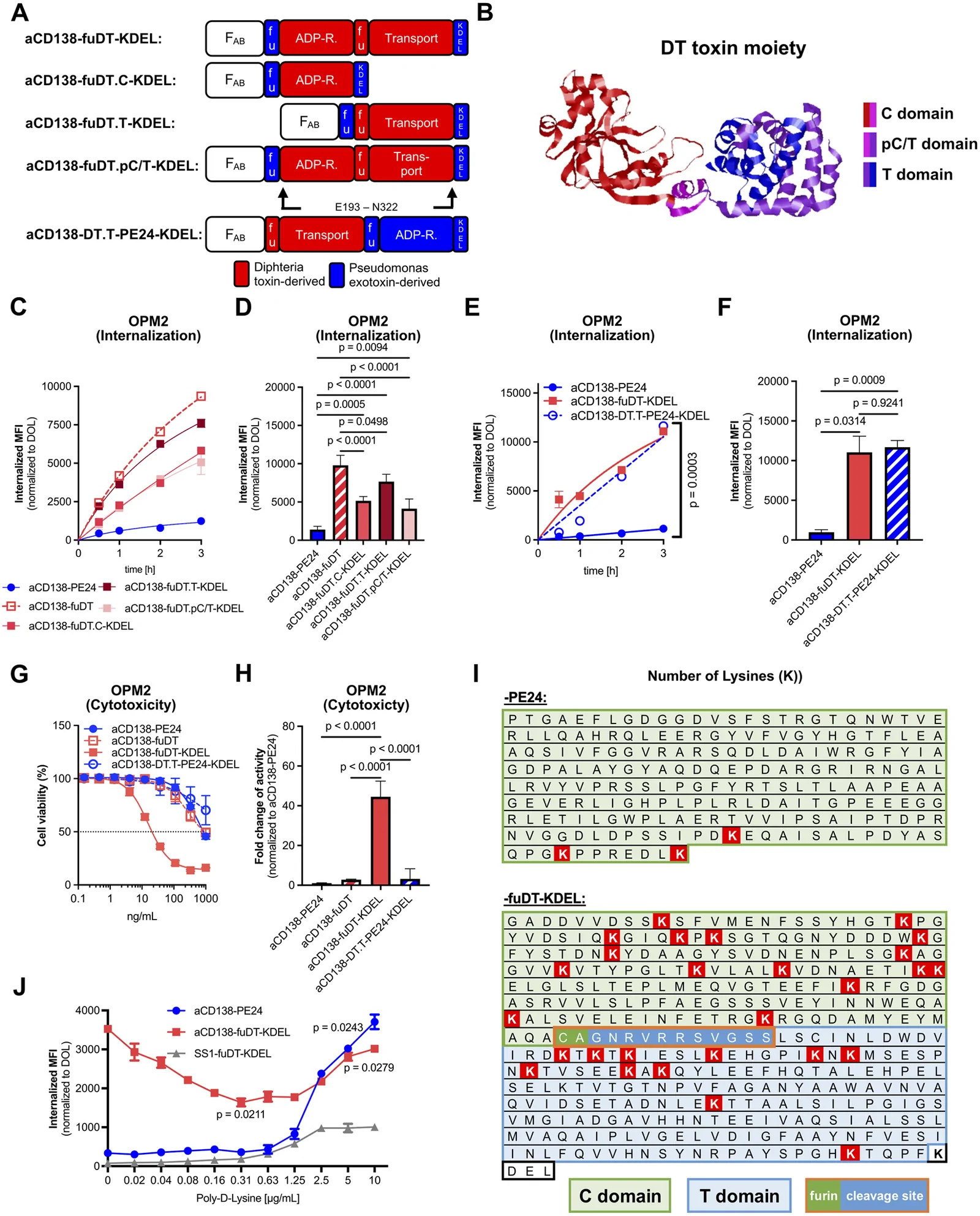
Role of the DT transport domain in enhanced internalization of fuDT-KDEL-based RITs. **(A)** Schematic representation illustrating the hypothesis-driven restructuring of DT- and PE-domains. **(B)** Ribbon structure of the DT toxin moiety based on 1F0L (https://doi.org/10.2210/pdb1F0L/pdb), with colors indicating the truncation of the DT-domain into three subdomains. The C-domain is highlighted in red and pink, while the T-domain is shown in blue and purple. The pink and purple ribbons specifically indicate the regions of the C and T domains selected for the overlap of the aCD138-fuDT.pC/T-KDEL. **(C)** Internalization assay on OPM-2 cells incubated with 14 nM of AF647-labeled aCD138-PE24, aCD138-fuDT, aCD138-fuDT.C-KDEL, aCD138-fuDT.T-KDEL, or aCD138-fuDT.pC/T-KDEL over time. Internalized molecules after surface stripping were measured via flow cytometry and normalized to the DOL in mole dye per mole RIT. Experiments were performed in triplicates; a representative of three similar results is shown. **(D)** Internalized MFI as seen in **(C)** at 3 h of incubation and normalized to the DOL. **(E)** Internalized MFI in OPM-2 cells incubated with 14 nM of AF647-labeled aCD138-PE24, aCD138-fuDT-KDEL, or aCD138-fuDT.T-PE24-KDEL for 0.5 h, 1 h, 2 h, and 3 h at 37 °C. Flow cytometry analyses were performed, and the results were normalized to the DOL. **(F)** Internalized MFI as seen in **(E)** at 3 h of incubation and normalized to the DOL. **(G)** Cell viability of OPM-2 cells treated with a 3-fold serial dilution of aCD138-PE24, aCD138-fuDT, aCD138-fuDT-KDEL, or aCD138-fuDT.T-PE24-KDEL for 72 h at 37 °C and measured by flow cytometry after 7-AAD staining. **(H)** Fold change of activity normalized to aCD138-PE24 was calculated for OPM-2 cells using IC_50_ values pooled from three independent experiments with error bars as SD. Activity is defined as the inverse IC_50_. For **(C,E,G)**, non-linear regression analyses were performed to highlight the progression of internalization or cell death depending on the time of incubation or RIT dose. Each data point represents the mean of two technical replicates, with error bars as SD. **(C,E,G)** are representative for three independent experiments, respectively, while for **(D,F,H)**, data from three independent experiments were pooled. Statistical significances were determined either by two-way ANOVA combined with Šídák’s multiple comparisons test **(E)** or by ordinary one-way ANOVA combined with Tukey’s multiple comparisons test **(D,F,H)**. **(I)** fuDT-KDEL-based RITs harbor a notably higher amount of lysines in their toxin moiety compared to that in PE24-based immunotoxins. **(J)** Treatment of OPM-2 cells with a 2-fold serial dilution of PDL for 1.5 h at 37 °C, co-incubated with 14 nM of AF647-labeled aCD138-PE24, aCD138-fuDT-KDEL, or SS1-fuDT-KDEL as an unspecific control. Statistical significances show the differences between 0 μg/mL PDL and 0.31 μg/mL or 10 μg/mL PDL and were calculated by two-way ANOVA combined with Tukey’s multiple comparisons test.

A faster internalization of DT than PE had not been described previously, so we next analyzed which part of DT was critical for this advantage. We dissected DT into three partially overlapping protein fragments based on the available structural models: the ADP-ribosylating C-terminal domain (fuDT.C), the transport domain (fuDT.T), and a partial overlap of the two domains (fuDT.pC/T) (Figures 3A,B). Testing the domain-dependent rate of internalization, we found all DT-based variants internalized significantly faster than aCD138-PE24 in OPM-2 cells (Figures 3C,D). The DT.C and the DT.pC/T variants were internalized similarly faster than PE24, while the DT.T variant was the fastest and was only minimally but significantly slower than the lead toxin aCD138-fuDT.T-KDEL (Figure 3D). These data indicated that the T-domain was the most relevant for enhanced internalization. However, the C-domain and the pC/T-domain containing RITs internalized faster than PE24, supporting that not a domain-specific amino acid sequence but rather a DT-spanning attribute was responsible for enhanced internalization. We then designed a control protein, in which we added the DT-T domain to PE24 with the goal of improving PE-internalization (aCD138-DT.T-PE24-KDEL) (Figure 3A). Notably, aCD138-DT.T-PE24-KDEL outperformed the internalization of aCD138-PE24, underscoring the ability of the T-domain of DT to partially rescue the comparably low internalization rate of PE24-based RITs (Figure 3E). Three hours after the start of internalization, approximately 10-fold more aCD138-fuDT-KDEL and aCD138-fuDT.T-PE24-KDEL were internalized compared to the PE24 variant (Figure 3F). Despite the enhanced internalization of aCD138-DT.T-PE24-KDEL, the hybrid RIT did not enhance cytotoxicity against OPM-2 cells (Figures 3G,H), indicating that an additional step following internalization was needed for the higher efficacy of aCD138-fuDT-KDEL. As all fragmented DT-based RITs showed an elevated uptake in OPM-2 cells, we instead assumed that PE performed poorly. Because the T-domain rescued PE24 internalization, we hypothesized that the much higher number of positively charged lysines within the C- and the T-domains of DT as compared to PE24 might cause more efficient internalization (Figure 3I). To block the interactions of DT-lysines with potential binding partners, we competitively added increasing concentrations of Poly-D-Lysine (PDL) to perturb the binding equilibrium. aCD138-PE24 and aCD138-fuDT-KDEL were co-incubated with PDL for 30 min at 37 °C before being added to OPM-2 cells with various concentrations of PDL (Figure 3J). After 90 min of incubation, the remaining surface-bound molecules were washed off, and the cells were analyzed by flow cytometry. SS1-fuDT-KDEL served as the control for PDL-effects on target-independent internalization as OPM-2 cells do not express mesothelin. Compared to that with no PDL, internalization of aCD138-fuDT-KDEL gradually decreased with increasing presence of PDL (Figure 3J). In line with the increasing internalization of unspecific SS1-fuDT-KDEL from 1.25 μg/mL of PDL, internalization of aCD138-PE24 was substantially enhanced and reached similar levels as aCD138-fuDT-KDEL by increasing the PDL concentrations (Figure 3J). The substantial dose-dependent reduction in the internalization rate of aCD138-fuDT-KDEL upon the increase of PDL, which was not observed for aCD138-PE24, may indicate that the lysine residues in DT contribute, at least in part, to the enhanced internalization of DT-based immunotoxins.

### 3.4 Higher internalization does not always correlate with higher cytotoxicity

**FIGURE 4:**
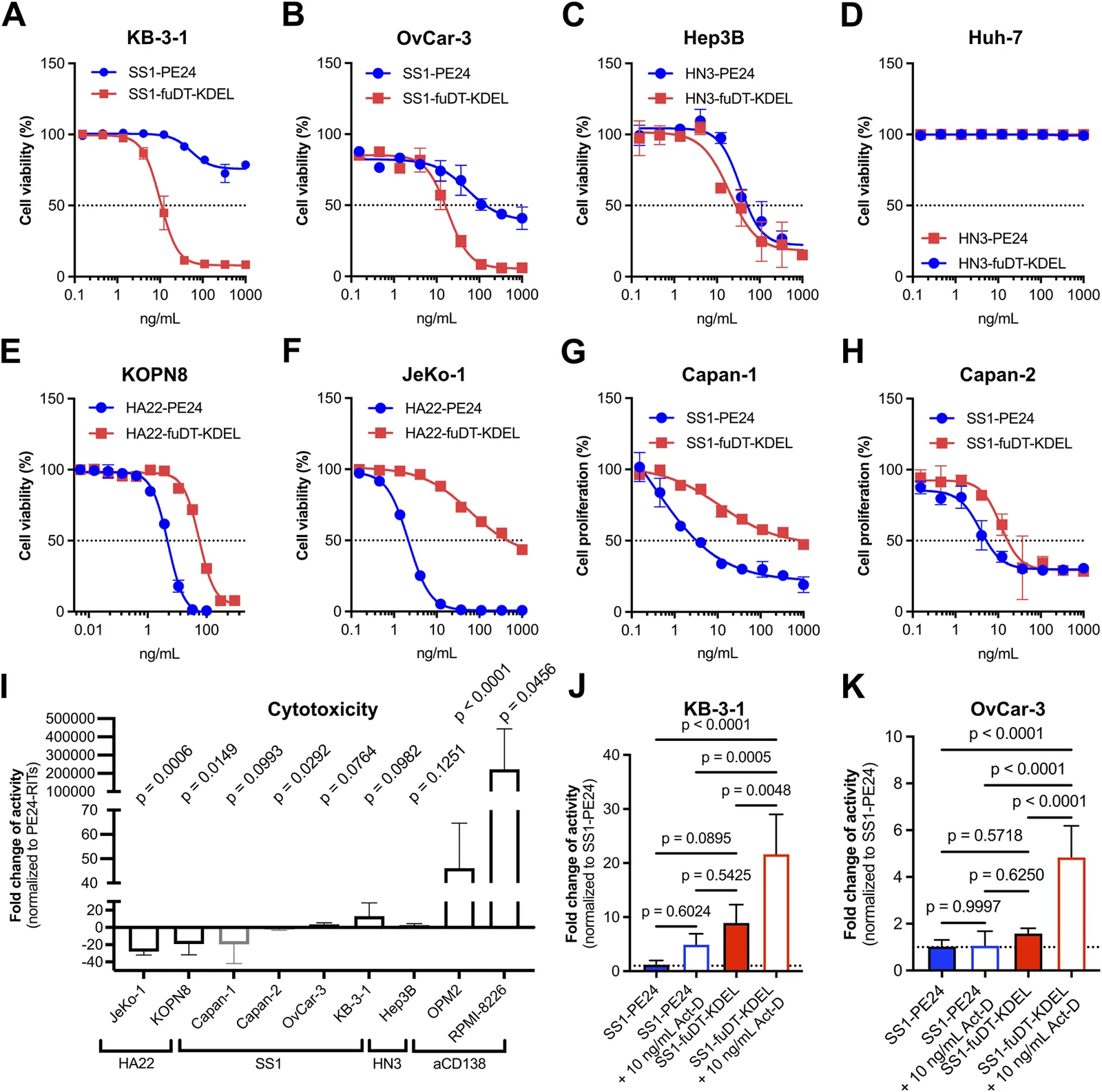
Cytotoxic activity of the fuDT-KDEL fusion protein depends on the target. Viability of KB-3-1 **(A)**, OvCar-3 **(B)**, Hep3B **(C)**, Huh-7 **(D)**, KOPN8, **(E)** and JeKo-1 **(F)** cells treated with indicated PE24 or fuDT-KDEL-based RIT. After staining with 7-AAD, cells were analyzed via flow cytometry. Cell proliferation of Capan-1 **(G)** and Capan-2 **(H)** treated with SS1-PE24 or SS1-fuDT-KDEL was measured by WST-8 assay. Non-linear regression analyses were performed to highlight the progression of cell death or proliferation arrest depending on the RIT dose; each dot represents the mean of two technical replicates, with error bars as SD. Each graph is representative of three independent experiments. **(I)** Fold change of activity of fuDT-KDEL-based RITs normalized to PE24-based RITs calculated for the respective cells using IC_50_ values pooled form three independent experiments, with error bars as SD. Activity is defined as the inverse IC_50_. Statistical significances were calculated by ratio paired t-tests. **(J,K)** Cell viability of KB-3-1 or OvCar-3 cells incubated with SS1-PE24 or SS1-fuDT-KDEL with or without 10 ng/mL of actinomycin D (Act-D). Viability was determined using 7-AAD by flow cytometry. Data are pooled from four (KB-3-1) and six (OvCar-3) independent experiments, and statistical significances were calculated by ordinary one-way ANOVA combined with Tukey`s multiple comparison test. Error is shown as SD.

To evaluate whether enhanced internalization of fuDT-KDEL immunotoxins translated into increased therapeutic activity, cytotoxicity assays were conducted. In line with MM cell lines (Figure 1I; Supplementary Figure S5D), the fuDT-KDEL toxin demonstrated more efficient killing of target cells compared to their PE24 counterparts for SS1-based immunotoxins against KB-3-1 and OvCar-3 cells (Figures 4A,B). A non-significant trend toward higher efficacy was observed in GPC3-targeted immunotoxins against Hep3B (Figure 4C). Neither fuDT-KDEL nor PE24 achieved cell death in Huh-7 liver cancer cells (Figure 4D). In contrast, PE-based anti-CD22 immunotoxins were more cytotoxic against B-cell malignancy cell lines KOPN8 and JeKo-1 than fuDT-KDEL-based immunotoxins (Figures 4E,F). Notably, neither SS1-PE24 nor SS1-fuDT-KDEL induced cell death within 3 days of incubation time in the pancreas cancer cell lines Capan-1 and Capan-2 (Supplementary Figure S10). In cells that were not killed by the immunotoxin, the impact of both RITs was assessed by measuring cell proliferation using WST-8 assays. Cell growth inhibition by SS1-PE24 was greater than by SS1-fuDT-KDEL in both Capan-1 and Capan-2 cells (Figures 4G,H). Representative cytotoxicity of fuDT-KDEL compared with PE24 was 30-fold lower against JeKo-1 (CD22), similar against Hep3B (GPC3), 13-fold higher against KB-3‐1 (mesothelin), and more than 40-fold higher against MM cell lines OPM-2 or RPMI-8226 (Figure 4I; Supplementary Table S2; Supplementary Figures S11A-G). In summary, the overall efficacy of the novel fuDT-KDEL-based RIT compared to its PE24 counterpart was dependent on the target antigen and the target cell line.

Since the efficacy of fuDT-KDEL was lower when targeting mesothelin than the CD22 counterparts, we aimed to test substances that are known to enhance immunotoxin intoxication in combination with the novel toxin (Cerise et al., 2019; Lin et al., 2023; Liu et al., 2016). We chose actinomycin D because it enhanced immunotoxin SS1-PE38 particularly strongly and because it supported the induction of cell death at the mitochondria, which is a common denominator of DT- and PE-based immunotoxins *in vitro* (Cerise et al., 2019). Actinomycin D on its own was cytotoxic against KB-3-1 with an IC_50_ of 20.95 ng/mL (Supplementary Figure S11H). In line with the previous findings, a sub-IC_50_ dose of 10 ng/mL of actinomycin D enhanced both SS1-PE24 and SS1-fuDT-KDEL (Figure 4J). The IC_50_ of 43.4 ng/mL for SS1-PE was reduced to 13.7 ng/mL, and the IC_50_ of SS1-fuDT-KDEL of 8.597 ng/mL was reduced to 2.736 ng/mL by the addition of sub-lethal doses of actinomycin D (Supplementary Figure S11I). The cytotoxicity of SS1-fuDT-KDEL was 7-fold stronger than that of SS1-PE24, which was enhanced 17-fold on average by actinomycin D (Figure 4J). Enhanced cytotoxcitiy by actinomycin D was reproduced in OvCar-3 (Figure 4K). Compared to SS1-PE24, SS1-fuDT-KDEL was 1.5-fold more active on average, which was increased to 4.6-fold higher cytotoxicity over SS1-PE24 by 10 ng/mL actinomycin D.

## 4 Discussion

When developing a novel PE-based RIT against CD138 on multiple myeloma, the unexpectedly inefficacious immunotoxin prompted the development of a novel PE‐DT-fusion toxin moiety. Once the antibody fragment including the PE furin-cleavage site was moved N-terminally of DT instead of the physiological C-terminal binding domain, the toxin demonstrated similar affinity but a superior internalization rate compared to that with PE-based toxins against various targets and tumor entities. In addition, only when the ER retention sequence ‘KDEL’ was added, the cytotoxicity was markedly enhanced against some but not all tumor entities tested. Mechanistically, the enhanced internalization of DT may, at least in part, be dependent on the effects of the positively charged lysine residues, which are largely absent in PE. However, this remains a tentative hypothesis based on indirect evidence and requires further validation.

The consistently higher internalization rate of the fuDT-KDEL-based RIT was interesting as it was target-, cell line-, and cancer entity-independent. This indicated a broadly relevant mechanism underlying the superiority in internalization. By fragmentation of DT, we unveiled the pivotal role of the pore-forming T-domain of DT in mediating the internalization of recombinant immunotoxins. Interestingly, the complete truncation of the translocation domain of PE does not harm but improves the function of PE-based RITs, so the positive effect on internalization by the T-domain is likely DT-specific (Hansen et al., 2010; Weldon et al., 2013). Notably, the catalytic or partially overlapping toxin moiety increased internalization, supporting the notion that not a specific receptor but a more unspecific effect increased the internalization rate of DT. This was further substantiated by the hybrid immunotoxin aCD138-DT.T-PE24-KDEL, an additional fusion of PE24 and DT.T, which increased the internalization rates of PE to similar levels as our optimized fusion toxin. Taking a closer look at the T-domain of DT, we noticed the high abundance of positively charged lysines (Figure 3H). The few lysines in PE-based immunotoxins are critical components for the activity of RITs (Ammon et al., 2022). In line with the critical role of lysines in DT for enhanced internalization, competition assays with PDL reduced aCD138-fuDT-KDEL internalization in a dose-dependent manner. Notably, PDL-coated proteins can be internalized unspecifically, which is thought to be caused by the positive charge of the coated proteins that enables easier passing of the negatively charged cell membrane more easily (Siow et al., 2018). The dose-dependent reduction of the fuDT-KDEL indicates a possible explanation for enhanced internalization because the lysine residues of the toxins facilitate transport over the negatively charged cell surface (Chen et al., 2016; Chen et al., 2009). The reduced number of lysines such as in PE, on the other hand, is believed to prevent proteasomal degradation in the cytosol by downregulating ubiquitination upon unfolding when PE must transit the translocon in a possibly at least partially unfolded way (Ammon et al., 2022; Worthington and Carbonetti, 2007). As such, a smaller sized T-fragment may further balance the rate of higher internalization and cytoplasmic degradation, which might lead to additional improvements of cytotoxicity for PE-based immunotoxins (Goulet and Atkins, 2020; Schmidt and Wittrup, 2009). Intriguingly, only after the addition of the KDEL-motive, improved internalization was translated into meaningfully enhanced cytotoxicity. These data from aCD138-targeting in MM supports previous data showing that typically much more DT than PE is internalized but only a small fraction of active DT escapes lysosomal degradation (Dorland et al., 1979; Saelinger et al., 1976). With this in mind, trafficking of the DT-KDEL variant is possibly re-routed toward the ER, which, according to this hypothesis, may prevent entrapment and degradation of the toxin in the endosomes (Pirie et al., 2011). However, we stay short of providing direct proof of this hypothesis due to the limitations of available methods. Intriguingly, all the approved and many of the potential preclinical DT-based immunotoxins are fusion proteins in which a cytokine replaces the natural binding domain of DT and are not constructed with dsFvs or Fabs (Alewine et al., 2015; Pastan et al., 2006; Wayne et al., 2014; Pemmaraju et al., 2019). For both our main immunotoxin and the supplementary DT-EB10 construct, N-terminal fusion of DT to the C-terminus of an antibody led to the loss of affinity, which we interpreted predominantly as dysfunction of the antibody moiety as the protein did not compete for binding with an intact PE-based immunotoxin. Because the placement of DT C-terminally restored binding, we suggest that the critical parts of the antibody may sterically be hindered or that refolding was impaired so the antibody turned defective. In line, no relevant antibody-DT constructs have been reported as of today. Therefore, our construct may also present an interesting fusion partner for other antibody-based DT immunotoxins.

Because we lack the respective data due to the lack of protein binding, we cannot rule out that the DT toxin function could also be impaired within the DT-aCD138 construct. The T-domain of DT comprises nine ɑ-helices (Choe et al., 1992; Ladokhin et al., 2021; Ghatak et al., 2015). Upon receptor binding, the C-terminal helices TH8 and TH9 are the first to insert into the endosomal membrane, followed by TH5–TH7 (Ladokhin et al., 2021). Together, these helices form a pore-like structure that facilitates the transition of the C-domain from the endosome into the cell cytoplasm (Choe et al., 1992; Ladokhin et al., 2021; Ghatak et al., 2015). The DT-aCD138 could thus block trafficking of DT and, therefore, the toxicity. In contrast, N- and/or C-terminal fusion of the staphylococcal protein ZZ to the DT T-domain did not perturb any structural changes that are important for protein folding or function (Chenal et al., 2002). However, this was only analyzed when using the T-domain as a membrane anchor, and no internalization processes were considered (Chenal et al., 2002). The more open structural rearrangement in fuDT-KDEL RITs may, therefore, further enhance internalization due to faster pore-formation, in addition to the high density of positively charged lysine residues that might promote rapid attraction to the negatively charged cell membrane.

Previous findings indicated a direct connection between faster internalization and higher cytotoxicity, as anti-CD22 RITs are internalized faster than those targeting CD19, leading to elevated activity of CD22 RITs (Du et al., 2008). A key feature of CD22 is its routing into the recycling compartment, a specialized trafficking compartment that is ideal for shuttling large amounts of ligand into a target cell (Du et al., 2008; Grant and Donaldson, 2009; O’Reilly et al., 2011). This contrasts with classic endocytosis, which is typically achieved when the growth factors are engaged by antibodies that cross-link the receptor (Moody et al., 2015). Supporting the relevance of trafficking pathways for monovalent immunotoxins, it should be noted that the intracellular signaling pathways of CD19 and CD22 also differ. CD22-directed internalization into the recycling compartment may be particularly favorable for PE (Du et al., 2008; Alderson et al., 2009; Müller et al., 2017; Kreitm and an, 2019; O'Reilly et al., 2011). Thus, distinct internalization depends not only on the toxin moiety but also on the target receptor engaged. In line, our fusion toxin did not always demonstrate superior cytotoxicity compared to the conventional PE-based immunotoxins, despite its enhanced internalization rate. Mesothelin is a GPI-anchored protein and can be internalized in endosomes and by pinocytosis. The internalization route varies according to the targeted cell (O'Reilly et al., 2011). In line, we find mesothelin-targeting of the fusion toxin in pancreatic cancers to be less active than PE24, while it was superior in KB-3-1 and OvCar-3. Following our observations, we would speculate that the increased cytotoxicity of the fuDT compared to that of PE in MM may be due to the internalization route of CD138. The observation that enhanced internalization does not always translate into higher cytotoxicity indicates that the target choice plays a critical role. Further mechanistic studies are required to dissect the hypothesis of target receptor dependence into possibly distinct internalization compartments. Finally, empiric testing may be necessary to define the best toxin for each individual cancer.

Another factor influencing the performance of RITs is the antigen density expressed by the analyzed cancer cell lines and tumor entities (Supplementary Figure S7J) (Chu et al., 2024; Gao et al., 2015; Hagemann et al., 2019; Ha et al., 2013; Lee et al., 2012; Yu et al., 2019). A possibly positive correlation between antigen density and the affinity of our immunotoxins may explain enhanced internalization. For example, HN3-based RITs bound to Hep3B cells exhibit a 22-fold higher MFI than those bound to Huh-7 cells, which may be explained by the 27-fold higher antigen density reported for Hep3B cells (Gao et al., 2015). Similar correlations appear to apply to CD138- and mesothelin-targeted constructs (Supplementary Figure S7) and are also reported for other immunotoxins such as those binding to HER2 (Hou et al., 2016). Numerous studies analyzing specific tumor-targeting agents have highlighted the importance of antigen density and the potential heterogeneity of expression across tumor cells and patients (Chu et al., 2024; Ramakrishna et al., 2019; Lazzerini et al., 2020; Li et al., 2022). Li et al. proposed that in addition to being tumor-associated, an ideal immunotoxin target should exhibit high antigen expression and a high internalization rate that is independent of the binding of immunotoxins (Li et al., 2022). In the context of anti-CD22 CAR T-cells, increasing the CAR affinity proved less effective than upregulating CD22 antigen density on the target cell surface (Ramakrishna et al., 2019). Likewise, mesothelin expression levels correlated closely with the therapeutic response of an anti-mesothelin antibody‐drug conjugate (Lazzerini et al., 2020). In contrast, Wayne et al. reported no apparent dependence of moxetumomab pasudotox on CD22-expression, which may have been biased by a possible role of domain-II in the process (Müller et al., 2018; Wayne et al., 2017).

Even though we proposed several hypotheses to explain why the internalization of our novel fuDT-KDEL RITs appears enhanced than that of PE24 RITs, the precise mechanisms and intracellular trafficking pathways of these constructs remain to be elucidated. The working hypotheses that the positive charge of fuDT-KDEL RITs influences internalization and that RITs are rerouted to the ER upon the addition of KDEL requires validation by knock-out screens or charge neutralization strategies in future studies. Comparing RIT internalization in the presence of positively, neutrally, or even negatively charged polymers may further clarify the role of positively charged lysines. Acetylation of lysine residues, which neutralizes their charge and has been reported to affect membrane interactions, represents another potential strategy to confirm our charge hypothesis (Fritz and Hirschey, 2013; Okada et al., 2021). Moreover, characterization of the intracellular trafficking of the RIT with a focus on endosomal escape, lysosomal degradation, and visualization in the endosome could provide additional mechanistic insights toward elucidating the distinct trafficking routes.

There are several limitations to our study in addition to the described lack of direct proof for some of the mechanisms underlying the enhanced cytotoxicity of the CD138 directed fusion immunotoxins, including a direct proof for the exact intracellular re-routing of the novel protein. To date, we have demonstrated an *in vitro* potential design strategy for a lead candidate, which provides a framework for future immunotoxin design. However, successful clinical translation will require comprehensive *in vivo* evaluations in suitable models, including target specificity assessments and on-target and off-tumor toxicities. Off-target toxicities in antigen-negative cells were also not analyzed extensively *in vitro*. Furthermore, the RIT translation is limited due to the complex bacterial production, including refolding and endotoxin removal (Kreitman, 2009; George et al., 2021; Chen et al., 2009). In addition, the high immunogenicity of the drugs and the reported low efficacy in mesothelin-expressing cancers further reduce the likelihood of clinical translation (Pastan et al., 2006; Hagerty et al., 2020; Hollevoet et al., 2015). However, immunogenicity may be less relevant in hematologic cancer than in solid tumors as the immune system is more severely impaired by the treatment but also by the underlying disease, leading to delayed anti-drug immune responses and higher clinical efficacy (Lutz et al., 2024; Kreitman et al., 2005; Pelzl et al., 2025).

Despite those limitations, we show, for the very first time, that efficacious antibody-directed targeting of diphtheria-based toxins can be achieved by N-terminally placed antibody fragment in combination with the PE-furin-cleavage site, which then allows for highly superior internalization of the antibody‐toxin fusion protein compared with that of PE-based immunotoxins. By the addition of the ER-retention signal KDEL, the toxin achieves superior cytotoxicity, at least when directed against CD138 of multiple myeloma. This novel immunotoxin design justifies further preclinical testing with the long-term goal of potential translation in multiple myeloma.

## Data Availability

The original contributions presented in the study are included in the article/Supplementary Material; further inquiries can be directed to the corresponding author.
