# Correction: Novel PE-DT-fusion toxin enhances internalization and target-dependently restores the cytotoxicity of recombinant immunotoxins

**DOI:** 10.3389/fphar.2026.1979795

**Published:** 2026-09-07

**Authors:** Claudia Fischer, Kerstin Wendland, Anna Ammon, Franziska Gsottberger, Lisa Mellenthin, Srdjan Petkovic, Andreas Mackensen, Fabian Müller

**Affiliations:** 1 Department of Internal Medicine 5-Hematology and Oncology, Friedrich Alexander University Erlangen-Nuremberg (FAU) and Universitätsklinikum Erlangen, Erlangen, Germany; 2 Bavarian Center for Cancer Research (BZKF), Erlangen, Germany; 3 Laboratory of Translational Immuno-Oncology, Department of Biomedicine, University and University Hospital Basel, Basel, Switzerland; 4 Deutsches Zentrum für Immuntherapie (DZI), Friedrich Alexander University Erlangen-Nuremberg and Universitätsklinikum Erlangen, Erlangen, Germany

**Keywords:** cytotoxicity, diphtheria toxin, internalization, *pseudomonas* exotoxin, recombinant immunotoxins, solid and hematological cancers, vesicular trafficking

The research was generously supported by a grant from the STAEDTLER-foundation awarded to FM which was erroneously omitted. The updated Funding statement appears below:

The author(s) declared that financial support was received for this work and/or its publication. This work was supported in part by a generous donation of the Geis family, by funds of the interdisciplinary center for clinical research of the University of Erlangen (IZKF, grant-# ELAN-P002 and ELAN-P085 to FM), and by the Deutsche Forschungsgemeinschaft (DFG, German Research Foundation, grant-# MU 3619/2–1) to FM. FM was supported by the German Cancer Aid (DKH, grant No 0113695) and by the Bundesministerium für Bildung und Forschung (BMBF-#01EO2105). The research was generously supported by a grant from the STAEDTLER-foundation awarded to FM.

The original version of this article has been updated.

