# Supplementary material for: Novel PE‐DT-fusion toxin enhances internalization and target-dependently restores the cytotoxicity of recombinant immunotoxins
Source: Front Pharmacol. 2026 Aug 14;17:1842007. doi: 10.3389/fphar.2026.1842007 (PMC13522408; doi:10.3389/fphar.2026.1842007)
Supplement: Supplementary file 1 [file DataSheet1.pdf]

Supplementary Material

Novel PE-DT-fusion toxin enhances internalization and cytotoxicity of recombinant immunotoxins in solid and hematological cancers

Claudia Fischer, Kerstin Wendland, Anna Ammon, Franziska Gsottberger, Lisa Mellenthin, Srdjan Petkovic, Andreas Mackensen, Fabian Müller

Supplementary Table 1: Overview of the subculturing procedures for investigated cell lines.

Cell line	Medium
OPM2 (RRID: CVCL_1625)<br>KOPN8 (CVCL_1866, LMB, NCI, NIH)<br>JeKo-1 (CVCL_1865, DSMZ, ACC 553)	RPMI-1640<br>+ 10% FBS<br>+ 2 mM L-glutamine<br>+ 100 U penicillin<br>+ 100 mg streptomycin
OvCar-3 (CVCL_0465)	RPMI-1640<br>+ 25% FCS<br>+ 0.01 mg/mL Insulin<br>+ 100 U penicillin<br>+ 100 mg streptomycin
Capan-1 (CVCL_0237)	IMDM<br>+ 20% FBS<br>+ 100 U penicillin<br>+ 100 mg streptomycin
Capan-2 (CVCL_0026)	McCoy's 5A Medium<br>+ 10% FBS<br>+ 100 U penicillin<br>+ 100 mg streptomycin
KB-3-1 (CVCL_2088, DSMZ, ACC 158)<br>Hep3B (CVCL_0326)<br>Huh-7 (CVCL_0336)	DMEM<br>+ 10% FBS<br>+ 100 U penicillin<br>+ 100 mg streptomycin

Supplementary Table 2: IC50 values of PE24-based and fuDT-KDEL-based recombinant immunotoxins across different target cell lines. Mean IC50 values from three independent experiments per cell line are presented in ng/mL. Statistical significance was assessed using a ratio paired t.test comparing PE24- and fuDT-KDEL-based RITs.

Antibody fragment	Target	IC50 in ng/mL (PE24-based RITs)	IC50 in ng/mL (fuDT-KDEL-based RITs)	IC50 in ng/mL (fuDT-based RITs)	p-value
aCD138	OPM2	753.17	16.67	276.23	p = 0.0007
	RPMI-8226	1676064.97	6.49	194.80	p = 0.0597
HA22	JeKo-1	1.90	57.65		p = 0.018
	KOPN8	4.62	94.22		p = 0.0068
SS1	Capan-1	2.69	87.94		p = 0.0993
	Capan-2	3.26	8.32		p = 0.0252
	OvCar-3	102.43	28.72		p = 0.212
	KB-3-1	86.76	8.30		p = 0.002
HN3	HEp3B	31.38	14.18		p = 0.1081

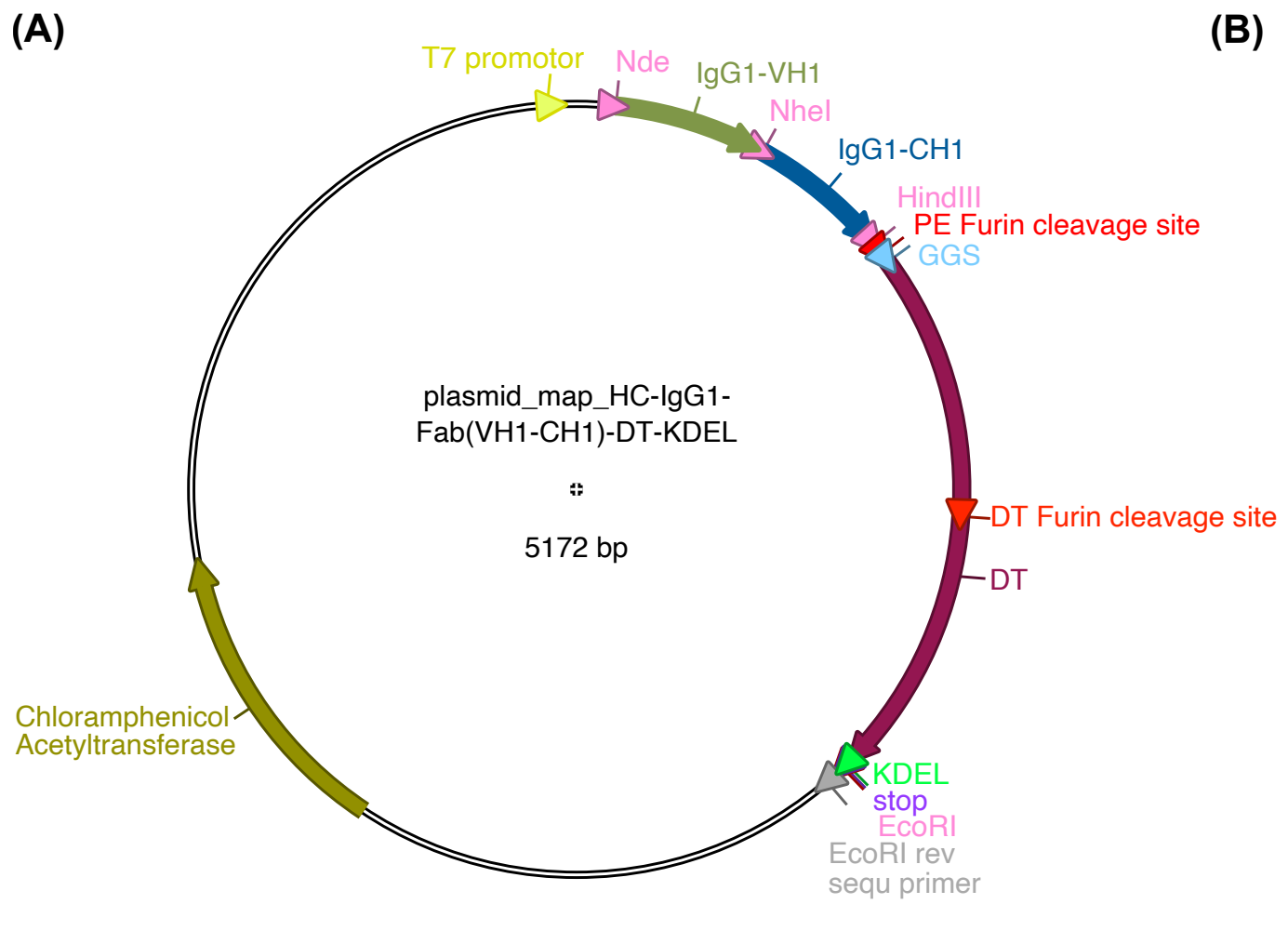

Suppl. Figure S1. Representative plasmid maps of light and heavy chains of recombinant immunotoxins. (A) Plasmid map of RIT heavy chain consisting of an IgG1 variable heavy (VH1) and constant heavy (CH1) chain and the toxin moiety (here: DT). **(B)** Plasmid map of RIT light chain consisting of a IgK variable (V1) and constant (C1) light chain.

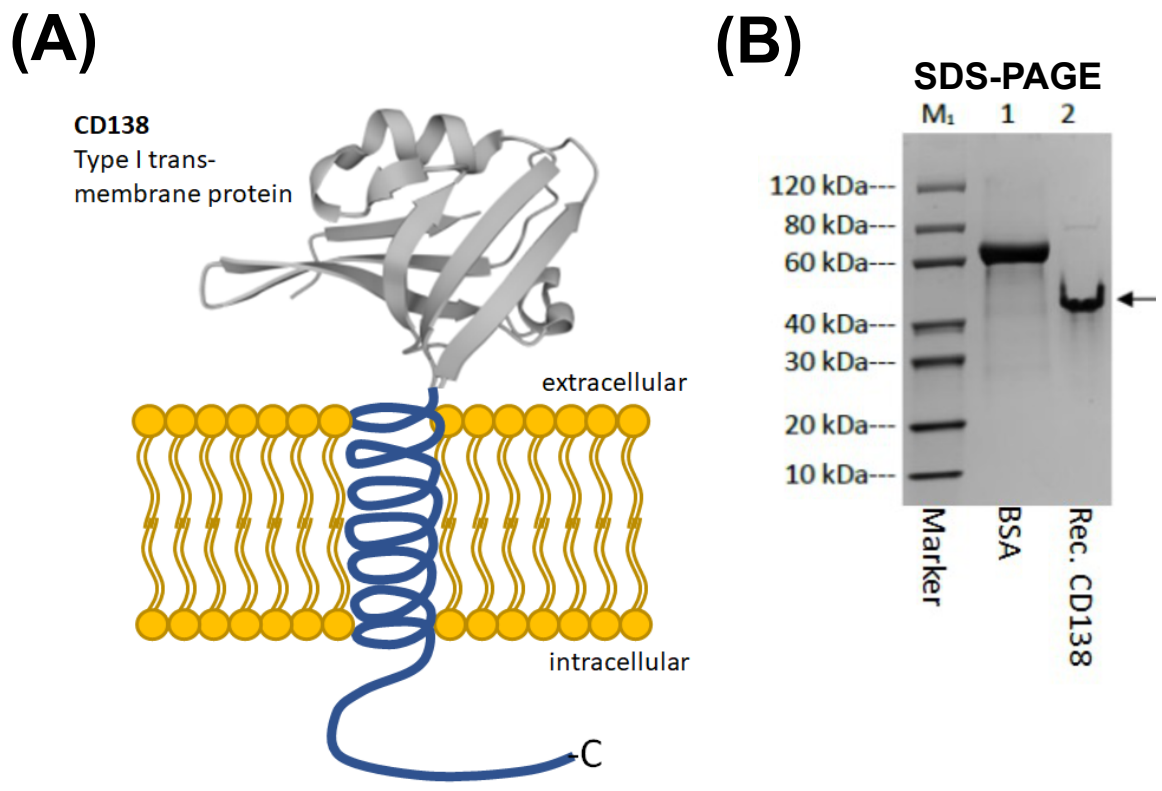

Suppl. Figure S2. Generation of anti-CD138 directed rabbit antibodies. (A) CD138 is a type I transmembrane protein consisting of an extracellular, a transmembrane and a cytoplasmic domain (Riccardi et al., 2024). **(B)** SDS-PAGE analysis confirming the purity of the recombinant (rec.) transmembrane domain of CD138 including an Fc-Tag used for purification (3 µg, lane 2). BSA serves as an additional molecular-weight marker in lane 1.

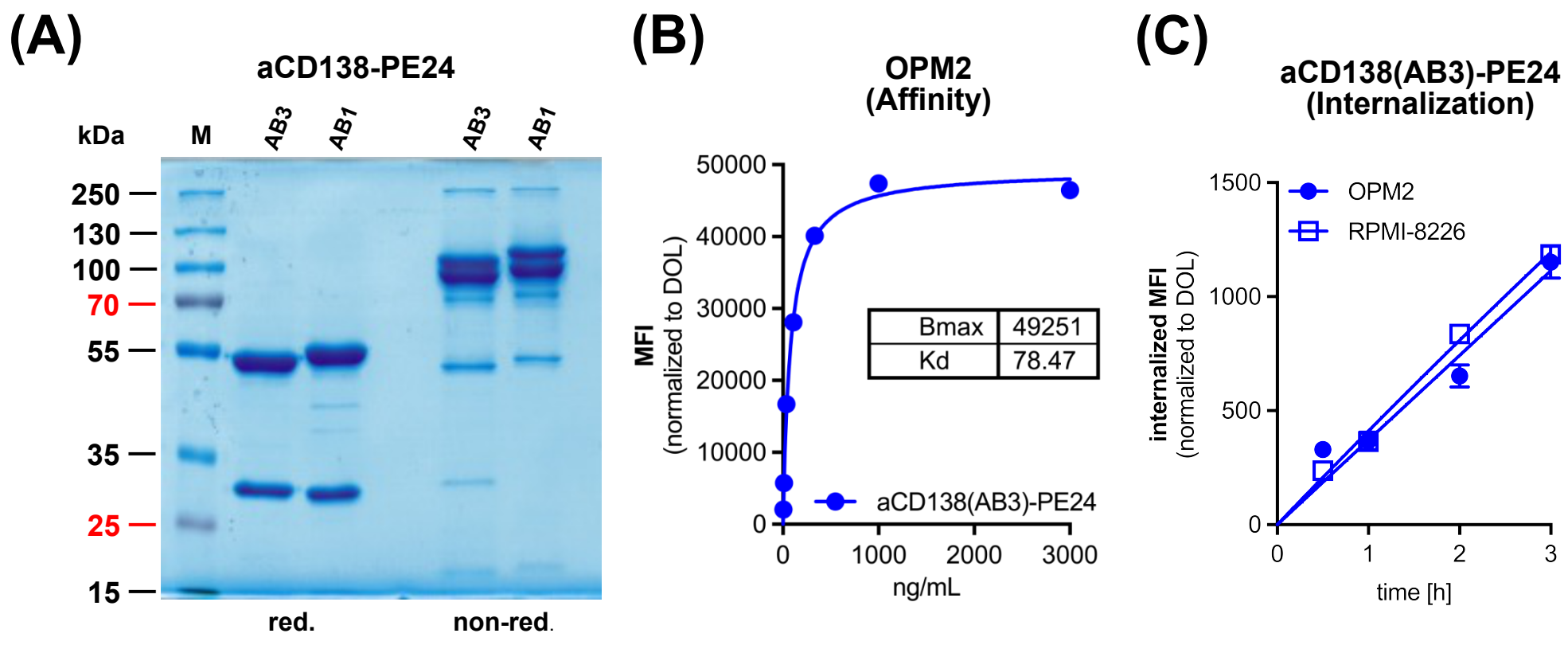

Suppl. Figure S3. Purity and performance of aCD138(AB3)-PE24 to multiple myeloma cells. (A) SDS-PAGE analysis of aCD138-PE24 RITs consisting either of AB1 (72.8 kDa) or AB3 (71.8 kDa) under reducing (left) and non-reducing (right) conditions. Both antibodies comprise antibody binding fragments (F_{AB}) harboring a HC (AB1: 49.3 kDa, AB3: 48.2 kDa) and LC (AB1: 23.5 kDa, AB3: 23.6 kDa). **(B)** MFI of OPM2 cells bound by aCD138(AB3)-PE24 after treatment with a 3-fold serial dilution of the RIT for 1 h at 4 °C. **(C)** Internalized MFI of OPM2 or RPMI-8226 cells incubated with 14 nM of AF647-labeled aCD138(AB3)-PE24 for 0.5 h, 1 h, 2 h and 3 h at 37 °C. Non-linear regression analyses were performed to depict the binding over time depending on dose (B) or the amount of internalization depending on time of incubation (C). Graphs are representative for four and three independent experiments, respectively.

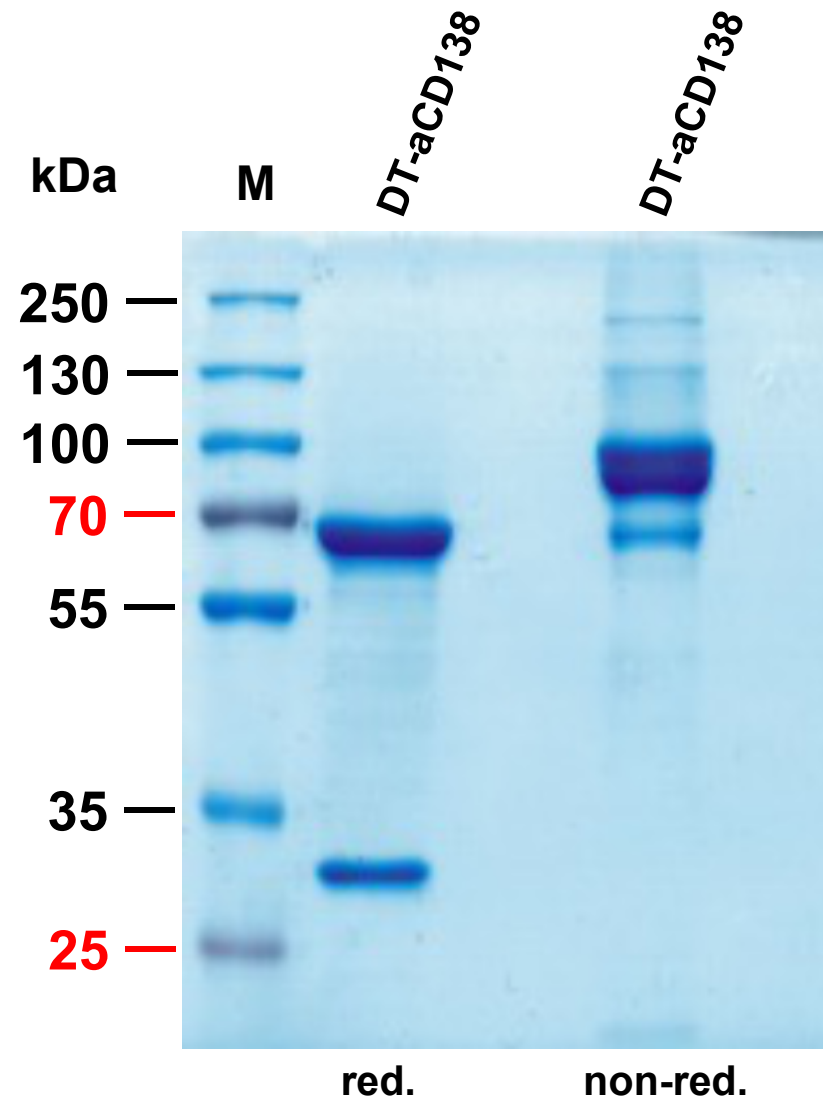

Suppl. Figure S4. Purity of DT-aCD138. SDS-PAGE analysis of DT-aCD138 (90 kDa) under reducing (left) and non-reducing (right) conditions. For the former, the RIT is separated into its LC (24 kDa) and HC (65 kDa).

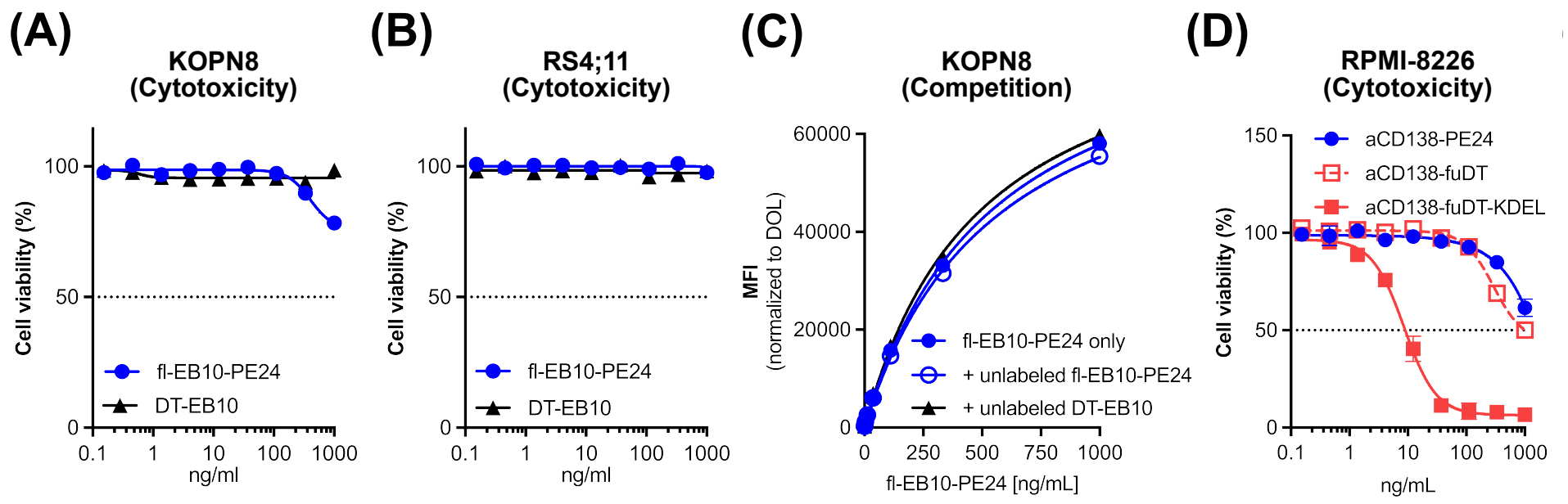

Suppl. Figure S5. Finding the structure for the novel, superior RIT. (A, B) Cell viability of KOPN8 or RS4;11 cells incubated with a 3-fold serial dilution of full length (fl)-EB10-PE24 or DT-EB10 for 72 h at 37 °C. Cells were analyzed after 7-AAD staining with flow cytometry. **(C)** MFI of KOPN8 cells treated for 1 h at 4 °C with a 3-fold serial dilution of AF647-labeled full fl-EB10-PE24 alone or competing with 80 ng/mL of either unlabeled fl-EB10-PE24 or unlabeled DT-EB10 for cell binding. **(D)** Cell viability of RPMI-8226 cells incubated with a 3-fold serial dilution of aCD138-PE24, aCD138-fuDT or aCD138-fuDT-KDEL for 72 h at 37 °C, measured by flow cytometry. Non-linear regression analyses were performed to highlight the progression of binding competition, cell death or internalization depending on the RIT dose. Each data point represents either one (A-C) or the mean of two (D) technical replicates with error bars as SD. (A-C) are representative for one and (D) for three independent experiments.

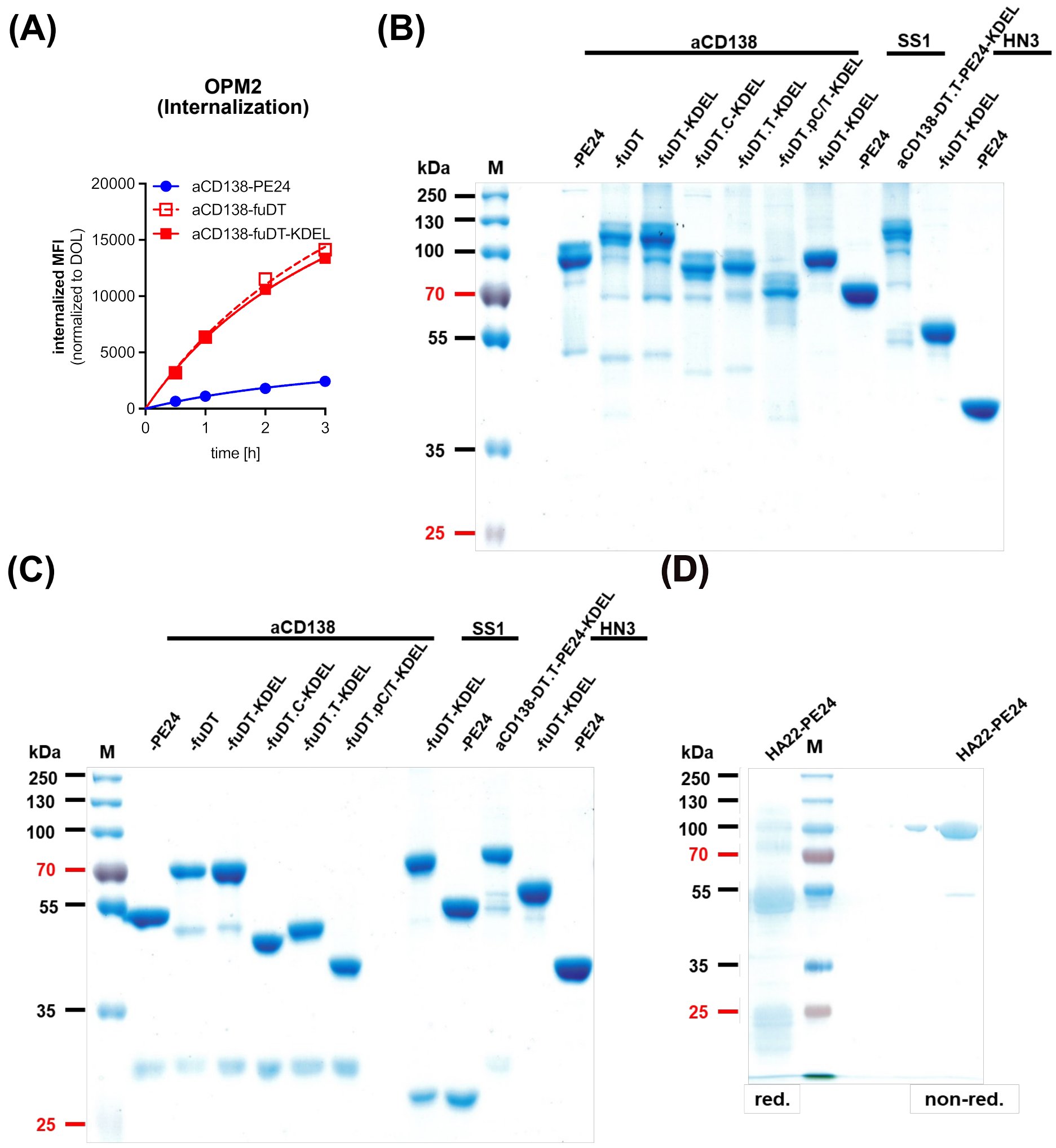

Suppl. Figure S6. Purity of fuDT-KDEL and PE24-based RITs. (A) Internalization on OPM2 cells incubated with 14 nM of AF647-labeled aCD138-PE, aCD138-fuDT or aCD138-fuDT-KDEL over time. Surface bound RIT was stripped and internalization was normalized to the DOL. Non-linear regression analyses were performed to highlight the progression of internalization over time. Each data point represents the mean of two technical replicates with error bars as SD. The graph is representative for three independent experiments. **(B)** SDS-PAGE analyses of RITs targeting CD138 (PE24: 72 kDa, fuDT: 91 kDa, fuDT-KDEL: 91 kDa, fuDT.C-KDEL: 69 kDa, fuDT.T-KDEL: 71 kDa, fuDT.pC/T-KDEL: 63 kDa, DT.T-PE24-KDEL: 94 kDa), SS1 (B, PE24: kDa, fuDT-KDEL: 91 kDa) and HN3 (B, PE24: 39 kDa, fuDT-KDEL: 58 kDa) under non-reducing conditions. **(C)** SDS-PAGE of RITs analyzed in (B) under reducing conditions separating the LCs (aCD138: 24 kDA, SS1: 23 kDa), and HCs (aCD138-PE24: 48 kDa, aCD138-fuDT(-KDEL): 67 kDa, aCD138-fuDT.C-KDEL: 45 kDa, aCD138-fuDT.T-KDEL: 47 kDa, aCD138-fuDT.pC/T-KDEL: 40 kDa, aCD138-DT.T-PE24-KDEL: 70

kDa, HN3-fuDT-KDEL: 58 kDa, HN3-PE24: 39 kDa, SS1-fuDT-KDEL: 68 kDa, SS1-PE24: 49 kDa). **(D)** SDS-PAGE of HA22-PE24 under reducing (LC: 24 kDa, HC: 50 kDa) and non-reducing (74 kDa) conditions.

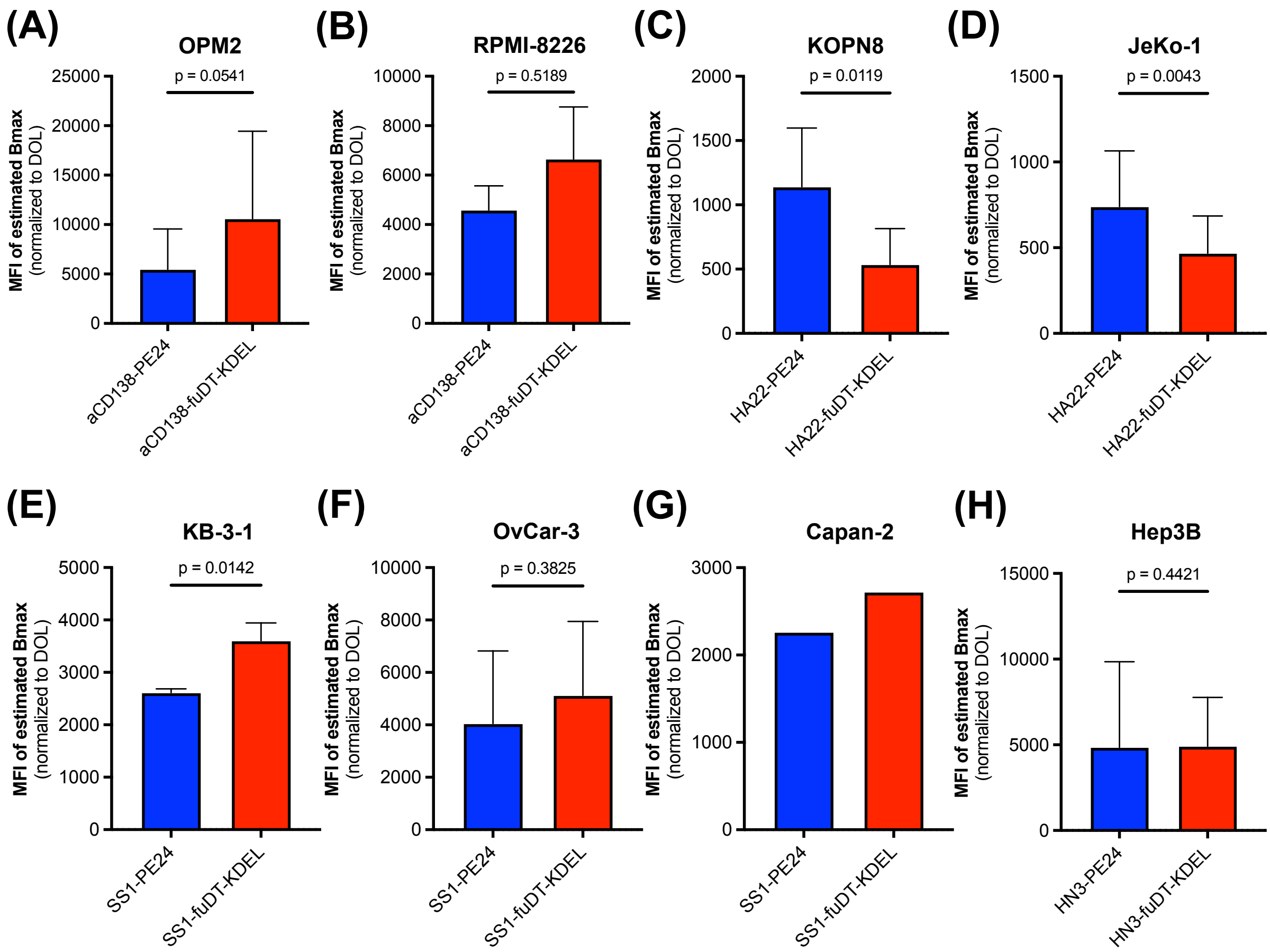

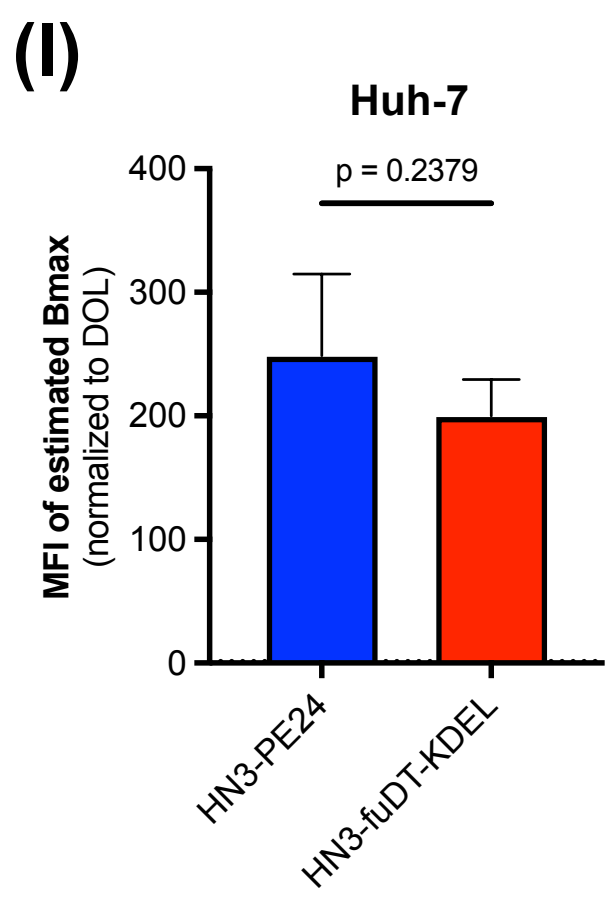

(J)

Cell Line	Cancer Entity	Target Antigen	Antigen density	Source
OPM2	MM	CD138	higher than RPMI-8226	https://doi.org/10.1182/blood-2019-127454
RPMI-8226	MM	CD138	lower than OPM2	https://doi.org/10.1182/blood-2019-127454
KOPN8	ALL	CD22	~16000 molecules/cell	10.1182/blood-2012-06-438002
JeKo-1	MCL	CD22	NA	
KB-3-1	endocervical adenocarcinoma	MSN	NA	
Capan-1	pancreatic ductal adenocarcinoma	MSN	438 molecules/µm^2	10.1016/j.nano.2012.01.005
Capan-2	pancreatic ductal adenocarcinoma	MSN	8191 molecules/cell	https://doi.org/10.1016/j.jcyt.2024.01.011
OvCar-3	ovarian serous adenocarcinoma	MSN	37877 molecules/cell	https://doi.org/10.1158/1078-0432.CCR-18-3476
Hep3B	HCC	GPC3	250000 molecules/cell	https://doi.org/10.1038/ncomms7536
Huh-7	HCC	GPC3	9000 molecules/cell	https://doi.org/10.1038/ncomms7536

Suppl. Figure S7. Affinity of novel fuDT-KDEL RITs to the investigated cell lines. MFI of maximum specific binding capacity (Bmax) of OPM2 **(A)**, RPMI-8226 **(B)**, KOPN8 **(C)**, JeKo-1 **(D)**, KB-3-1 **(E)**, OvCar-3 **(F)**, Capan-2 **(G)**, Hep3B **(H)** and Huh-7 **(I)** cells incubated for 1 h at 4 °C with AF647-labeled PE- or DT-based RITs. Results were acquired via flow cytometry and normalized to the DOL. Bmax was determined via non-linear regression analysis. Data are

cumulative from three independent experiments (exceptions: RPMI-8226: n = 2; Capan-2: n=1), and statistical significances were calculated by ratio paired t.test. **(J)** Overview of target antigen density on individual cell lines as found in literature. MM: Multiple Myeloma, MCL: Mantle Cell Lymphoma, ALL: Acute Lymphoblastic Leukemia, HCC: Hepatocellular carcinoma (Yu et al., 2019, Haso et al., 2013, Lee et al., 2012, Chu et al., 2024, Hagemann et al., 2019, Gao et al., 2015).

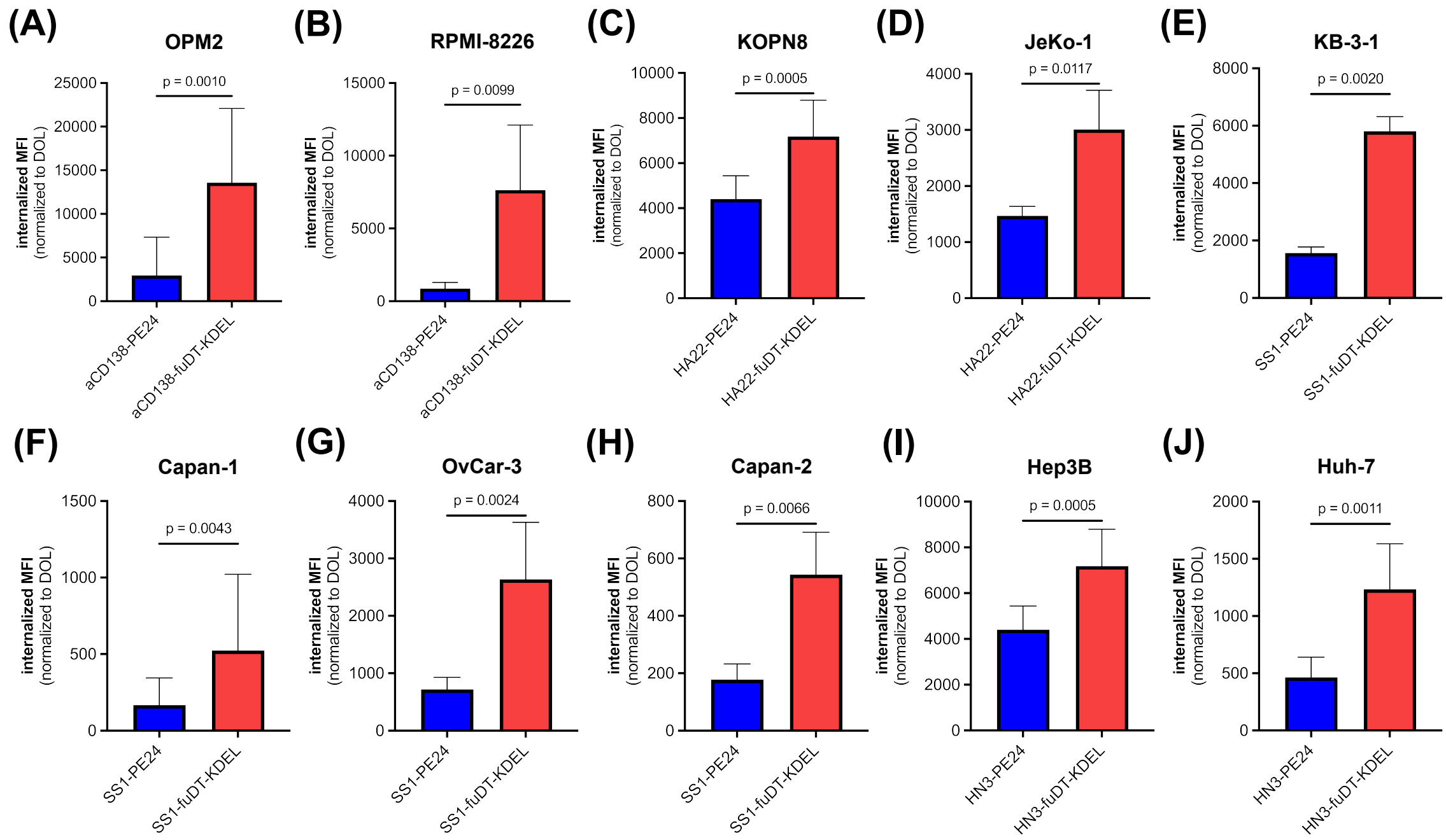

Suppl. Figure S8. Improved target-independent internalization of novel fuDT-KDEL-based immunotoxins. Internalized MFI of OPM2 **(A)**, RPMI-8226 **(B)**, KOPN8 **(C)**, JeKo-1 **(D)**, KB-3-1 **(E)**, Capan-1 **(F)**, OvCar-3 **(G)**, Capan-2 **(H)**, Hep3B **(I)** and Huh-7 **(J)** cells incubated for 3 h at 37 °C with indicated AF647-labeled PE- or DT-based RITs. Results were acquired via flow cytometry and normalized to the DOL. Data are cumulative from three independent experiments, and statistical significances were calculated by ratio paired t.test.

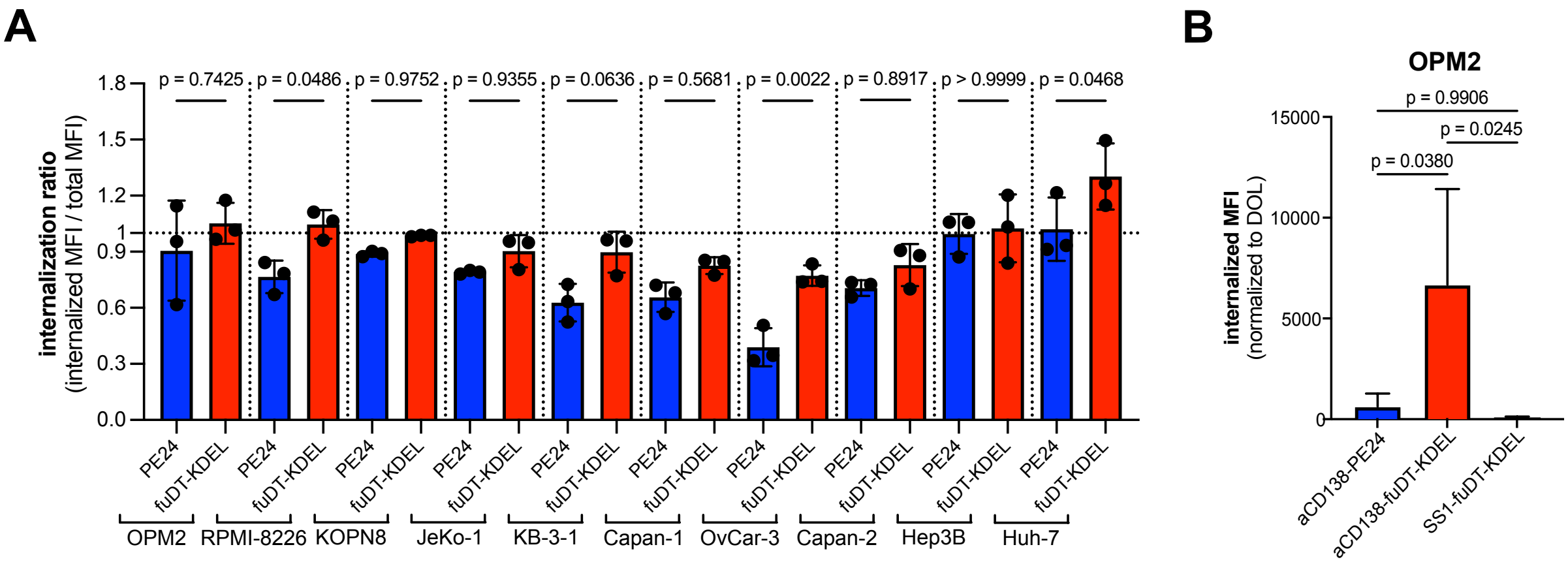

Suppl. Figure S9. Internalization ratio of PE24- and fuDT-KDEL-based immunotoxins. **(A)** Internalized MFI of RITs was divided by total MFI with the latter being defined by all surface bound and internalized signal. Each dot represents two technical replicates with overall data being derived from three independent experiments. **(B)** Internalized MFI of on- and off-target RITs incubated for 90 min at 37°C with OPM-2 cells. Data is derived from three independent experiments. Statistical significances were calculated by Ordinary one-way ANOVA combined with Šidák`s multiple comparison test.

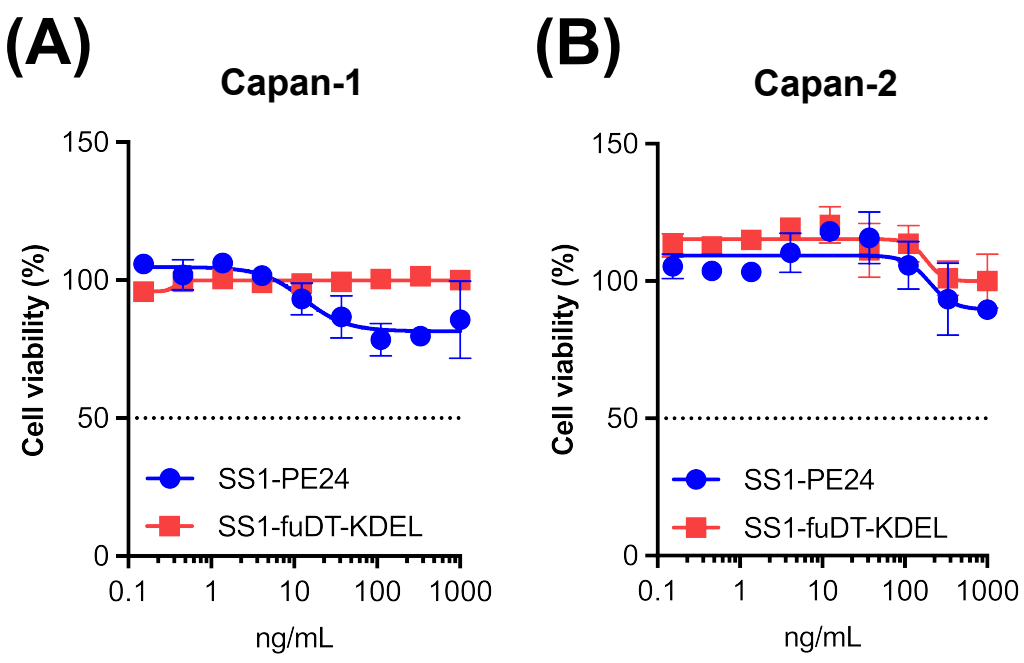

Suppl. Figure S10. Failure of the fuDT-KDEL-based RIT targeting mesothelin to induce cell death in pancreatic cancers. (A) Viability of Capan-1 cells incubated with a 3-fold serial dilution of SS1-PE24 or SS1-fuDT-KDEL for 72 h at 37 °C. **(B)** Viability of Capan-2 cells incubated with a 3-fold serial dilution of SS1-PE24 or SS1-fuDT-KDEL for 140 h at 37 °C. Non-linear regression analyses were performed to highlight the progression of cell death depending on the RIT dose; each dot represents the mean of two technical replicates with error bars as SD. Each graph is representative for three independent experiments.

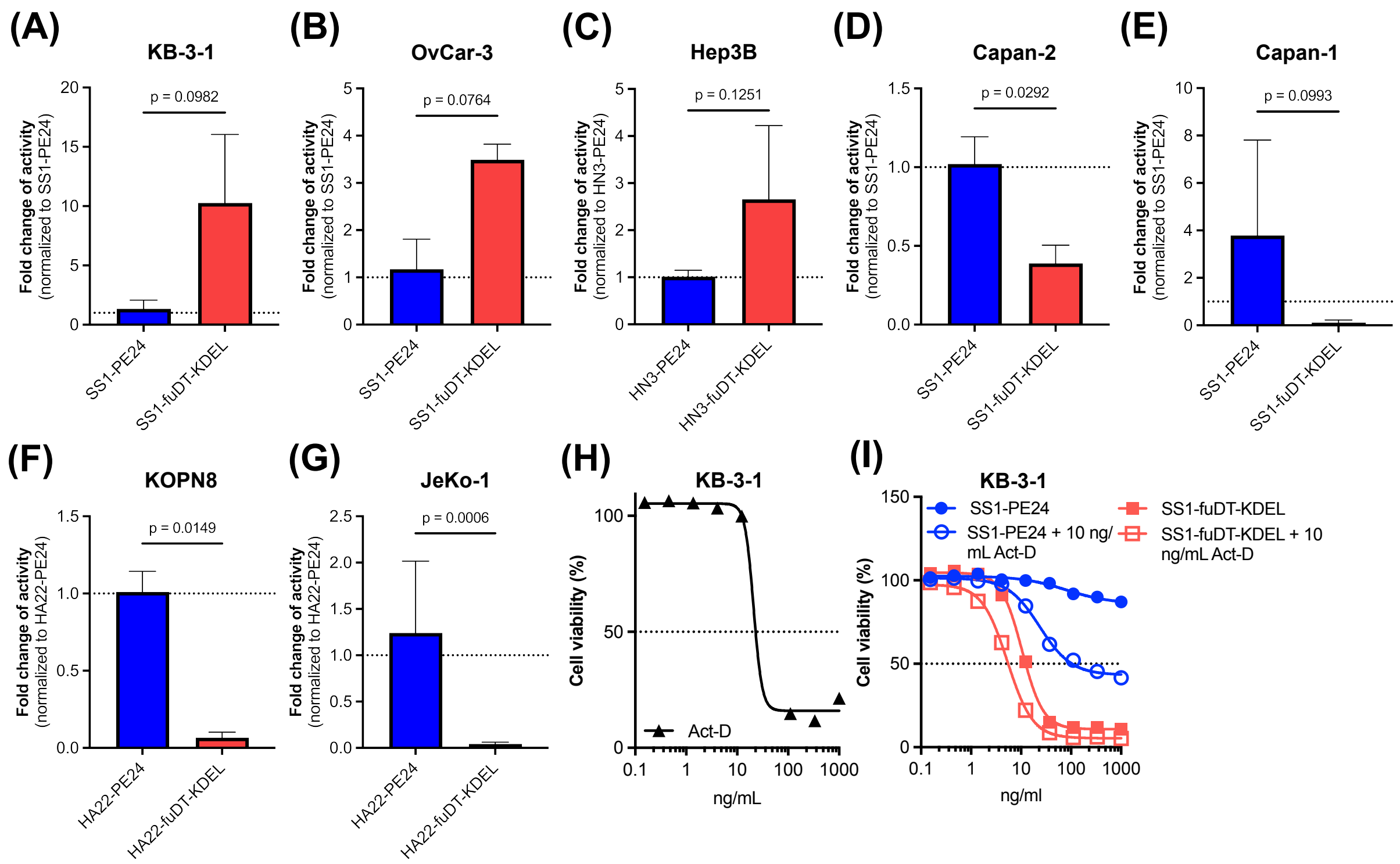

Suppl. Figure S11. Target-dependent cytotoxic activity of the novel fuDT-KDEL-based immunotoxins. Fold change of activity of PE24- or fuDT-KDEL-based RITs after 72 h incubation at 37 °C with KB-3-1 **(A)**, OvCar-3 **(B)**, Hep3B **(C)**, Capan-2 **(D)**, Capan-1 **(E)**, KOPN8 **(F)**, Jeko-1 **(G)** cells. The fold change was normalized to aCD138-PE24 and calculated using IC_{50}-values pooled from three independent experiments, with error bars as SD. Statistical significances were determined by Ordinary one-way ANOVA combined with Tukey‘s multiple comparisons test. **(H)** Cell viability of KB-3-1 cells incubated with a 3-fold serial dilution of Actinomycin D (Act-D), determined after 72 h at 37 °C using flow cytometry. **(I)** Cell Viability of KB-3-1 cells incubated with SS1-PE24 or SS1-fuDT-KDEL with or without 10 ng/mL of Actinomycin D for 71 h at 37 °C. (I) is representative for four independent experiments.

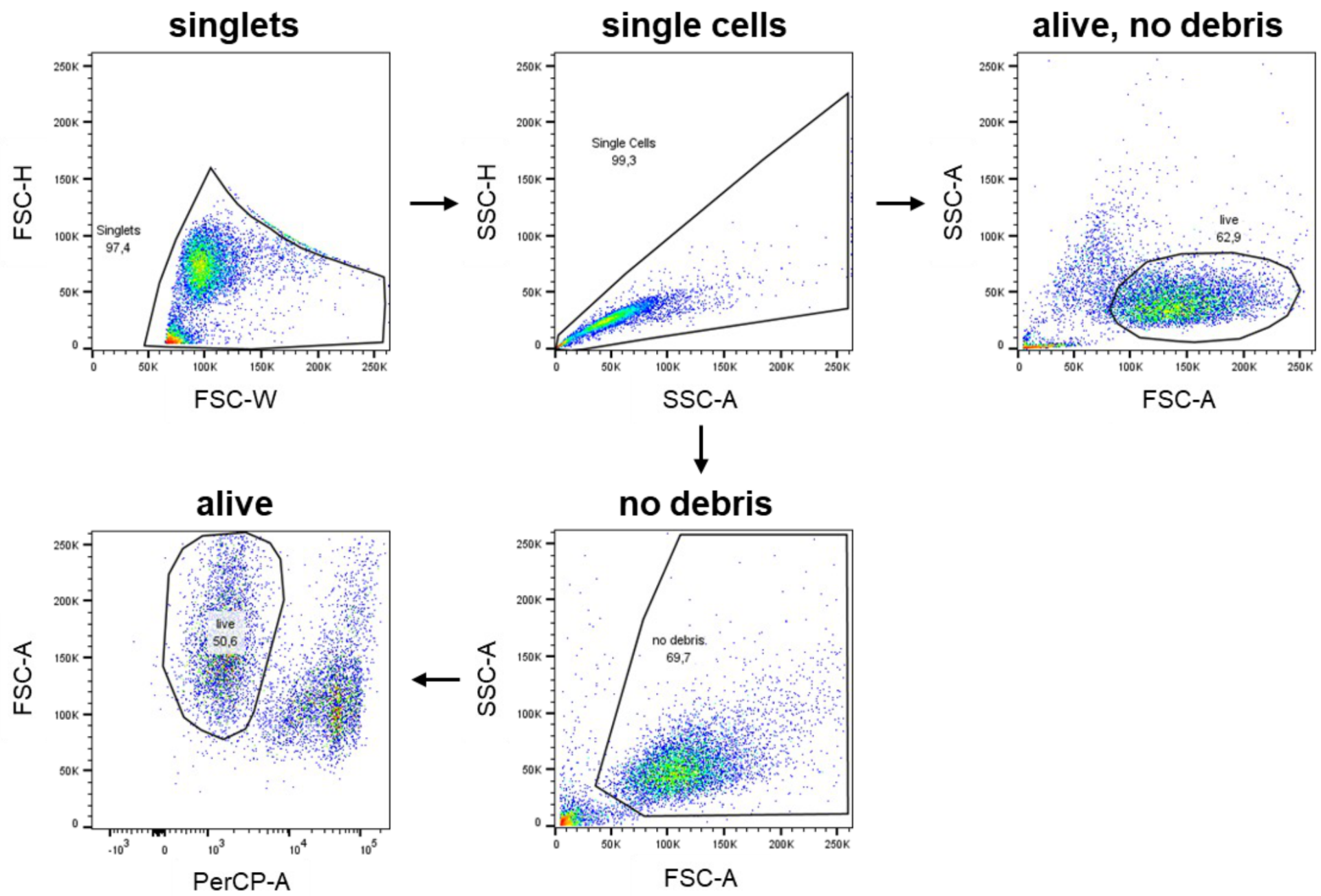

Suppl. Figure S12. Gating strategy for flow cytometric analyses. Gating of different cancer cell lines using BD FACSCanto™ II. Numbers indicate the percentage of cells within the gated population. Arrows indicate the order of the gating procedure.

References:

CHU, G. J., BAILEY, C. G., NAGARAJAH, R., LIANG, O., METIERRE, C., SAGNELLA, S. M., CASTELLETTI, L., YEO, D., ADELSTEIN, S. & RASKO, J. E. J. 2024. Mesothelin antigen density influences anti-mesothelin chimeric antigen receptor T cell cytotoxicity. *Cytotherapy,* 26**,** 325-333.

GAO, W., TANG, Z., ZHANG, Y. F., FENG, M., QIAN, M., DIMITROV, D. S. & HO, M. 2015. Immunotoxin targeting glypican-3 regresses liver cancer via dual inhibition of Wnt signalling and protein synthesis. *Nat Commun,* 6**,** 6536.

HAGEMANN, U. B., ELLINGSEN, C., SCHUHMACHER, J., KRISTIAN, A., MOBERGSLIEN, A., CRUCIANI, V., WICKSTROEM, K., SCHATZ, C. A., KNEIP, C., GOLFIER, S., SMEETS, R., URAN, S., HENNEKES, H., KARLSSON, J., BJERKE, R. M., RYAN, O. B., MUMBERG, D., ZIEGELBAUER, K. & CUTHBERTSON, A. S. 2019. Mesothelin-Targeted Thorium-227 Conjugate (MSLN-TTC): Preclinical Evaluation of a New Targeted Alpha Therapy for Mesothelin-Positive Cancers. *Clinical Cancer Research,* 25**,** 4723-4734.

HASO, W., LEE, D. W., SHAH, N. N., STETLER-STEVENSON, M., YUAN, C. M., PASTAN, I. H., DIMITROV, D. S., MORGAN, R. A., FITZGERALD, D. J., BARRETT, D. M., WAYNE, A. S., MACKALL, C. L. & ORENTAS, R. J. 2013. Anti-CD22–chimeric antigen receptors targeting B-cell precursor acute lymphoblastic leukemia. *Blood,* 121**,** 1165-1174.

LEE, K. H., GALLOWAY, J. F., PARK, J., DVORACEK, C. M., DALLAS, M., KONSTANTOPOULOS, K., MAITRA, A. & SEARSON, P. C. 2012. Quantitative molecular profiling of biomarkers for pancreatic cancer with functionalized quantum dots. *Nanomedicine: Nanotechnology, Biology and Medicine,* 8**,** 1043-1051.

RICCARDI, F., TANGREDI, C., DAL BO, M. & TOFFOLI, G. 2024. Targeted therapy for multiple myeloma: an overview on CD138-based strategies. *Frontiers in Oncology,* Volume 14 - 2024.

YU, T., LIN, L., WEN, K., XING, L., LIU, J., CHO, S.-F., HSIEH, P., RIZQ, O., LI, Y., YU, L., WU, X., MORELLI, E., XU, Y., AN, G., MUNSHI, N. C., QIU, L., ANDERSON, K. C. & TAI, Y.-T. 2019. Syndecan-1 Is Critical in ARF6-Dependent Macropinocytosis Driven By KRAS Mutation in the Pathophysiology of Multiple Myeloma. *Blood,* 134**,** 4371.
